# Fabrication of pure Bi_2_WO_6_ and Bi_2_WO_6_/MWCNTs nanocomposite as potential antibacterial and anticancer agents

**DOI:** 10.1038/s41598-024-58751-y

**Published:** 2024-04-25

**Authors:** Zeena R. Rhoomi, Duha S. Ahmed, Majid S. Jabir, Abdul Qadeer, Alaa B. Ismael, Ayman A. Swelum

**Affiliations:** 1 Applied Science Department, University of Technology-Iraq, Baghdad, Iraq; 2 Department of Cell Biology, School of Life Sciences Central South University, Changsha, China; 3 Department of Animal Medicine, Faculty of Veterinary Medicine, Zagazig University, Sharkia, Egypt; 4 Department of Animal Production, College of Food and Agriculture Sciences, King Saud University, Riyadh, Saudi Arabia

**Keywords:** Nanoflakes Bi_2_WO_6_, Bi_2_WO_6_/MWCNTs, Antibacterial, Liver cancer, Cytotoxicity, ROS, Apoptosis, Biological techniques, Cancer, Microbiology, Molecular biology

## Abstract

An essential research area for scientists is the development of high-performing, inexpensive, non-toxic antibacterial materials that prevent the transfer of bacteria. In this study, pure Bi_2_WO_6_ and Bi_2_WO_6_/MWCNTs nanocomposite were prepared by hydrothermal method. A series of characterization results by using XRD FTIR, Raman, FESEM, TEM, and EDS analyses, reveal the formation of orthorhombic nanoflakes Bi_2_WO_6_ by the addition of NaOH and pH adjustment to 7. Compared to pure Bi_2_WO_6_, the Bi_2_WO_6_/MWCNTs nanocomposite exhibited that CNTs are efficiently embedded into the structure of Bi_2_WO_6_ which results in charge transfer between metal ion electrons and the conduction or valence band of Bi_2_WO_6_ and MWCNTs and result in shifting to longer wavelength as shown in UV–visible and PL. The results confirmed that MWCNTs are stuck to the surface of the microflowers, and some of them embedded inside the Bi_2_WO_6_ nanoflakes without affecting the structure of Bi_2_WO_6_ nanoflakes as demonstrated by TEM. In addition, Pure Bi_2_WO_6_ and the Bi_2_WO_6_/MWCNTs nanocomposite were tested against *P. mirabilis* and *S. mutans.,* confirming the effect of addition MWCNTs materials had better antibacterial activity in opposition to both bacterial strains than pure Bi_2_WO_6_. Besides, pure Bi_2_WO_6_ and the Bi_2_WO_6_/MWCNTs nanocomposite tested for cytotoxicity against lung MTT test on Hep-G2 liver cancer cells, and flow-cytometry. Results indicated that pure Bi_2_WO_6_ and the Bi_2_WO_6_/MWCNTs nanocomposite have significant anti-cancer efficacy against Hep-G2 cells in vitro. In addition, the findings demonstrated that Bi_2_WO_6_ and Bi_2_WO_6_/MWCNTs triggered cell death via increasing ROS. Based on these findings, it appears that pure Bi_2_WO_6_ and the Bi_2_WO_6_/MWCNTs nanocomposite have the potential to be developed as nanotherapeutics for the treatment of bacterial infections, and liver cancer.

## Introduction

The photocatalyst semiconductors have garnered increasing interest recently as an antibacterial agent due to considerable chemical stability and broad-spectrum antimicrobial capabilities. The antibacterial effects of photocatalysts semiconductors on both negative and positive bacteria have been studied in many research^1–3^. Among a typical Aurivillius oxide, Bismuth tungstate (Bi_2_WO_6_) is a widely utilized Bi-based semiconductor due to its exceptional fundamental physical and chemical properties^4^. Bi_2_WO_6_ is a favorable photocatalyst with a narrow visible light absorption band (2.8 eV) for environmental treatment fields, friendly characteristics, chemically stable, surface Plasmon response (SPR), and low toxicity. It has also been demonstrated that Bi_2_WO_6_ exhibited photocatalytic degradation of organic pollutants and good potential for applications as an antibacterial agent^5–7^. In terms of controllable synthesis, Bi_2_WO_6_ nanoplates, nanosheets, nanorods, and nanoflowers can be made using the hydrothermal procedure, sol–gel technique, calcination, and electrodeposition. In addition, the hydrothermal procedure of Bi_2_WO_6_ frequently results in the creation of 2D nanostructure plates along the (001) plane as revealed in the previous papers^8–13^. Moreover, depending on how the experimental circumstances are adjusted, the strong electrostatic attraction between the WO_4_^2–^ and Bi^3+^ ions in an aqueous solution may cause diverse morphologies of Bi_2_WO_6_ crystals^14^. A wide range of Bi_2_WO_6_ micro/nanostructures, including nanoplates, nanoflower/sphere-like structures, and nanocage-like structures, were formed using a hydrothermal procedure^9,15^. On the other hand, using Bi_2_WO_6_ as a photocatalytic can be restricted because of the high recombination of photogenerated (e–h) pairs. To overcome these restrictions, active strategies like doping, coupling with carbon compounds, and forming nanocomposites can be achieved.

Furthermore, because of their unique properties, including a high surface-to-volume ratio and improved antibacterial activity as a result of their significant interaction with bacterial cells relative to micron-sized nanoparticles, nanocomposites are regarded as one of the most intriguing materials. Also, it was noted that nanoparticles are useful in several disciplines, including biomaterials^16^. Among these compounds, multi-walled carbon nanotubes (MWNTs) with a 1D dimension have grown throughout time as a result of unique physical and chemical characteristics in comparison to their bulk antecedents. Due to their large specific surface area and outstanding electronic conductivity, MWCNTs have received a lot of attention. Moreover, they are known for their exceptional mechanical, electrical, and thermal properties, making them promising candidates for energy storage. The incorporation of MWCNTs into other materials can enhance the storage related to increasing the stability of nanocomposites^17^. Besides, the reactive oxygen species (ROS) introduced on the surface of MWCNTs like COOH, OH, and O^2−^ are highly reactive which can convert the pollutants to non-toxic results. The bacterial fatality is attributed to the oxidative stress mechanism generated by (ROS) on the surface of nanostructures^18^. MWCNTs can professionally transfer the generated electrons and quickly separate the recombination of photoinduced carriers which enchased photocatalytic activity^19,20^. Besides, the adsorption capacity to pollutants is enhanced by MWCNTs' large surface area and ability to form an electron–electron interaction with pollutants which increases the removal effectiveness^20,21^. To reduce the rate of recombination of photoinduced charge carriers, adding another semiconductor material results in an increasing photocatalytic approach as reported by A.U. Khan et al. that provides Ag_2_S-ZnO/GO nanocomposite synthesized using sol–gel method and demonstrated effective photocatalysts and antifungal activates under visible light^22^. Recently, researchers found that the addition of carbon (like Mesoporous Carbon and graphene) to Bi_2_WO_6_ can enhance the efficiency of (e–h) separation, and enhance the photocatalytic activity of Bi_2_WO_6_ as reported by Chen et al. that provides MC/Bi_2_WO_6_ composite^23^ and Chuansheng et al., that provide Bi_2_WO_6_/GO nanohybrid as supported in photocatalysis and antibacterial applications^24^. In addition, the most significant use of carbon nanotubes in the medical and pharmaceutical fields is related to their high aspect ratio, significant surface area, functionalization of surface, and stability of size at the nanoscale^25–29^. In particular, multi-walled carbon nanotubes (MWCNTs) can damage the bacterial cell by damaging the DNA and then upsetting the bacteria enzymatic system exhibit special biomimetic characteristics with the internal cytoskeletal polymers, primarily with microtubules^30,31^. Furthermore, due to their unique features, carbon nanotubes are frequently used in cancer research. The ability of carbon nanotubes to absorb the radiation from light sources like (visible, UV, IR) laser and transform it into heat is one of its distinctive properties^32^. It is worth noting that the potential application of MWCNTs as antitumoral or anticancer drugs is merely based on their intrinsic features, employing unique cytotoxic mechanisms^33,34^.

This work aims to prepare Pure Bi_2_WO_6_ and Bi_2_WO_6_/MWCNTs nanocomposites using hydrothermal procedure and provide a basic investigation on the morphology, structure, and chemical content of the materials by using XRD, UV–visible, PL, FTIR, Raman and FESEM, TEM with EDS analysis. Furthermore, the antibacterial activity of samples against *Streptococcus mutans*, and *Proteus mirabilis* pathogens were tested using an agar well diffusion assay. Also, in this work, Pure Bi_2_WO_6_ and Bi_2_WO_6_/MWCNTs nanocomposites have been used as anticancer agents that inhabit cell replication of liver cancer cells.

## Materials and methods

### Materials

Bismuth nitrate pentahydrate Bi(NO_3_)_3_.5H_2_O (98% ACS) and Sodium tungstate dehydrate Na_2_WO_4_.2H_2_O (99%) were purchased from Sigma-Aldrich, Germany company, Hexadecyltrimethylammonium CTAB (98%), nitric acid (HNO_3_,65%) where purchased from CDH Company, India, and multi-walled carbon nanotubes (MWCNTs purity > 95 wt%; diameter 8–15 nm Grafton Company, USA) is used in this work. The entire experiment was conducted with deionized water.

### Synthesis of pure Bi_2_WO_6_ and Bi_2_WO_6_/ MWCNTs nanocomposite

Pure Bi_2_WO_6_ was formed by the hydrothermal technique. To begin, 80 mL of D.W. was dissolved with 2 mmol of Bi(NO_3_)_3_.5H_2_O, 1 mmol of Na_2_WO_4_.2H_2_O, and 0.05g of [CTAB]. The final solution was stirred using a magnetic stirrer at room temperature for 30 min and adjusted to pH 7. Then the white suspension solution was sealed into a 100 mL autoclave of Teflon lined. After being sealed, the autoclave was maintained for 16 h at 180 °C in an oven with convection. Then the autoclave was cooled to ambient temperature and the white precipitate was developed. Bi_2_WO_6_ was washed several times in distilled water and ethanol before being dried in an oven with convection for 10 h at 60 °C. The same process was utilized to form the Bi_2_WO_6_/MWCNTs nanocomposite. 0.4 g of functionalizing F-MWCNTs were added after 0.4 g of prepared Bi_2_WO_6_ had been dissolved in 50 mL of deionized water and stirred for one hour in a beaker using a magnetic stirrer. Then, to accomplish dispersion, the mixture was dissolved in 100 mL of deionized water using ultrasonication for two hours. After an hour, the solutions are mixed and stirred. While this was going on, 2 mL of sodium hydroxide (NaOH) with a concentration of 4 mol/L was thoroughly dissolved in distilled water by a magnetic stirred at 25 °C for 20 min. Moreover, the Bi_2_WO_6_/MWCNTs solution was continuously stirred as the (NaOH) solution was added dropwise (3 drops per minute) until the pH was adjusted to 7. The combination was placed in a stainless steel autoclave with a Teflon liner that held 150 mL and heated for 16 h at 180 °C. After that, the autoclave was cooled to room temperature. The formation solution was then dried in the oven with convention at 60 °C for 12 h after being rinsed with water and ethanol.

### Characterization

Using XRD diffractometer (XRD diffraction 6000, Shimadzu) and CuK radiation (λ = 1.542Å), current 30 mA and voltage 40kV. The structure of pure Bi_2_WO_6_ and Bi_2_WO_6_/MWCNTs nanocomposite samples were investigated. The recorded data fell between 10° and 60°. The average particle size (D) was estimated using Scherrer equation D = kλ/βcosθ, where k is a constant (0.89), β is the full-width half maximum (FWHM) and θ is Bragg law. FTIR spectrum (8400S, Shimadzu) was employed to analyze the boundary construction of the resulting samples. The samples were mixed with KBr powder and pressed to form the semitransparent pellets. Using a wavelength of about 250 nm, the Photoluminescence (PL) spectroscopy was carried out using (Cary Eclipse fluorescence model, Iran). The optical properties were performed using UV–visible spectroscopy (Shimadzu UV-1800 spectrophotometer) and using suspension with a concentration of 0.1mg/mL in DW at a wavelength ranging from 200 to 800 nm at room temperature. The Raman spectra of Bi_2_WO_6_ and Bi_2_WO_6_/MWCNTs nanocomposite were achieved in a range of (200–1800) nm (Raman spectroscopy, Takram N1-541). The samples absorbed laser light photons (λ = 633 nm as excitation source), which are then reemitted. The field emission scanning electron microscopy (FE-SEM, TESCAN, MIRA3) images of Bi_2_WO_6_ and Bi_2_WO_6_/MWCNTs samples were captured to observe their morphology. The sample reflects the electrons that are emitted from the filament, and either secondary electrons or backscattered electrons are used to create pictures. Furthermore, Energy-dispersive X-ray spectroscopy (EDS) was used to determine the composition elements of the samples. For more details to study the morphology of samples, transmission electron microscopy (TEM, Philips-EM-208S) was used. High energy electron beam transmitted was used through a very thin sample to image and analyze the nanostructures and nanocomposite materials with atomic-scale resolution. Besides scanning electron microscopy (SEM Apreo2, Thermo Fisher Scientific, USA) was carried out to morphology the performance of Bi_2_WO_6_ and Bi_2_WO_6_/MWCNTs products on bacterial cells. Besides, energy-dispersive X-ray spectroscopy (EDS) was used as a relatively simple but powerful technique for the elemental analysis of samples.

### Antibacterial activity assay

The antibacterial activity of Pure Bi_2_WO_6_ and Bi_2_WO_6_/MWCNTs nanocomposite was assessed by agar well diffusion assay against *P. mirabilis* as gram's negative and *S. mutans* as gram's positive bacterial strains. Initially, about 20 mL of Muller-Hinton (MH) agar was aseptically poured into sterile Petri dishes. The bacterial species were collected from their stock cultures using a sterile wire loop. After culturing the organisms, using the tips of a sterile micropipette, 6 mm-diameter wells were bored on the agar plates. Different concentrations (62.5, 125, 250 μg/mL) of the bacterial samples were cultivated at 37°C overnight on Muller-Hinton (MH) agar (HiMedia India) comprising pure Bi_2_WO_6_ and Bi_2_WO_6_/MWCNTs nanocomposite. The agar plates were photographed, and the inhibition zones were measured.

### Scanning electron microscopy (SEM) of bacteria permeability

The damages to the outer cell membrane permeability were recognized by monitoring the bacterial morphology on agar plates. As explained in the above section, pure Bi_2_WO_6_ and Bi_2_WO_6_/MWCNTs nanocomposite samples are taken in different concentrations. Bacterial cells of *P. mirabilis* as Gram's negative and *S. mutans* treated with pure Bi_2_WO_6_ and Bi_2_WO_6_/MWCNTs nanocomposite were centrifuged at 4000 rpm and washed three times using phosphate buffer solution (50 mM, pH = 7.3) as well as using untreated (control). Then thin smear of the suspension was spread on the glass slide and maintained at room temperature until dry. Slides samples were visualized with a Scanning Electron Microscopy (SEM).

### Crystal violet staining

*P. mirabilis* and *S. mutans* at concentration (1 × 10^6^ CFU/mL) were grown in 24-well plates and treated with pure Bi_2_WO_6_ and Bi_2_WO_6_/MWCNTs nanocomposite at concentrations 125 μg/mL for 24h. After that, the samples were washed with PBS and *P. mirabilis* and *S. mutans* adhered wells were stained with crystal violet (0.1%, Sigma, USA) after rinsing twice with D.W. To measure biofilm development, 0.2 mL of 95% ethanol was added to crystal violet stained wells and incubated for 2 h while being shaken. The optical density was then calculated at 595 nm.

### Investigation of bacterial biofilm metabolic activity

Biofilms were formed in glass tubes in the presence and absence of the pure Bi_2_WO_6_ and Bi_2_WO_6_/MWCNTs samples^35^. After incubation at anaerobic conditions at 37 °C and for 48 h, the biofilm suspension was stained with a Live/Dead stain Kit and analyzed by flow cytometry. Briefly, 10 μL of Syto9 (30 μM) was added for 10 min, and then, 10 μL of propidium iodide (500 μM) was added for 10 min; the samples were washed 2 times in PBS and centrifuged for 2 min at 2000 rpm. The sample with two stain components was excited at 488 nm, and the emission was registered using the FITC channel for Syto 9 (530/30) and, (670/LP) channel for propidium iodide. The results of biofilm cell viability were expressed in the percentage of untreated control cells.

### Cytotoxicity assay (MTT assay)

The cytotoxicity of the pure Bi_2_WO_6_ and Bi_2_WO_6_/MWCNTs nanocomposite was investigated by MTT assay. After cultivation overnight, Hep-G2 and REF cells were seeded into 96-well plates at a density of 1 × 10^4^ cells per well. After removing the growth medium and replacing it with 200 µL of new medium containing various concentrations of Bi_2_WO_6_ and Bi_2_WO_6_/MWCNTs nanocomposite at a concentration (25 µg/mL) for 24, 48 h^36^. Following a wash with PBS, the cells were subjected to a three-hour treatment with an MTT solution containing 2 mg/mL (Invitrogen, Carlsbad, CA). After that, the solution was drained out of each well, and then 100 µL of DMSO was added to each one. A microplate reader was utilized to determine each sample's absorbance at a wavelength of 492 nm^37^. The equation that was used to determine the rate of inhibition of cell growth, also known as the percentage of cytotoxicity, is as follows.

$$\mathrm{ytotoxicity\%}=\frac{{\text{A}}-{\text{B}}}{{\text{A}}}$$

where A represents the optical density of the control and B represents the optical density of the samples^38^.

### Acridine orange/ethidium bromide staining (AO/EtBr)

In 12-well plates, the Hep-G2 cells were collected and plated. Following a 24-h incubation period, the cells were exposed to Bi_2_WO_6_ and Bi_2_WO_6_/MWCNTs nanocomposite for 24 h. Following that, the cells were stained by10 µg/mL AO/EtBr for 2 min at 37 °C, and detected by the fluorescent microscope.

### Flow cytometry assay

To measure the production of ROS in cells, a flow cytometry test was utilized. Hep-G2 cells were seeded at a density of 1 × 10^6^ per well. Following overnight incubation, the cells were treated with Bi_2_WO_6_ and Bi_2_WO_6_/MWCNTs nanocomposite for 8 h. After that, a ROS probe (DCFH-DA) at a concentration of 15 µM was added to the new medium and incubated for another 30 min in the dark. The fluorescence intensity of the cells was measured using a flow cytometer. In addition, flow cytometry assay was used to measure mitochondrial dysfunction assay using a Rhodamine probe, and Mitochondrial Membrane potential using (JC-1) probe. The fluorescence intensity of the cells was measured according to the manufactured protocol.

### Apoptosis detection annexin V/PI assay

Cell apoptosis was investigated using a flow cytometry assay. Liver cancer cells (Hep-G2) were treated with Bi_2_WO_6_ and Bi_2_WO_6_/MWCNTs nanocomposite at a concentration (25 µg/mL). The cells were taken out and collected after 24 h. Cells were twice washed with cold PBS, and stained for 30 min with an Annexin V FITC and PI solution. The labeled cells were then evaluated using a flow cytometry assay.

### Statistical analysis

The resulting data are the outcomes of three independent experiments. Data are represented as mean ± SD. GraphPad Prism (7) was used to carry out the statistical analysis via the application of the one-way ANOVA analysis of variance. The difference between means was assessed by LSD, in which p ≤ 0.05 was considered significant. *p ≤ 0.05, **p ≤ 0.01, ***p ≤ 0.001, ****p ≤ 0.0001^39^.

## Results and discussion

### Structural, optical, and morphological studies

**Figure 1:**
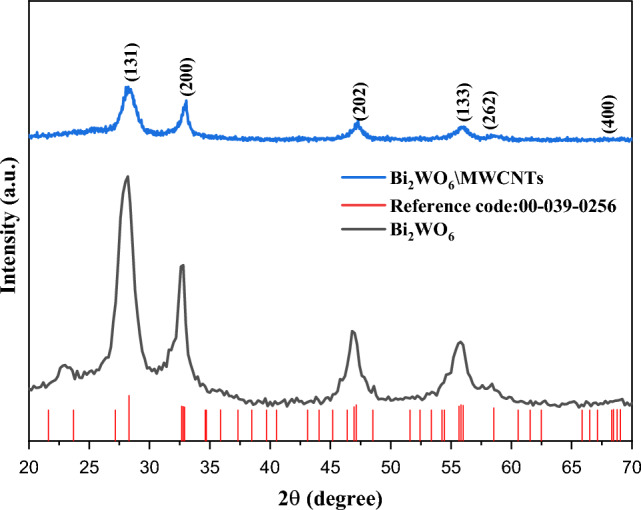
The XRD spectrum of Bi_2_WO_6_ and Bi_2_WO_6_/MWCNTs nanocomposites using hydrothermal method.

Figure 1, displayed XRD patterns of obtained pure Bi_2_WO_6_ and Bi_2_WO_6_/MWCNTs nanocomposite. Due to the sample crystallinity in Fig. 1 (black line), the pure Bi_2_WO_6_ displays high-intensity and narrow diffraction peaks in the XRD diffraction pattern. The diffraction peaks agree with the structure of orthorhombic Bi_2_WO_6_ (JCPDS card no. 39-0256). The values of the Bi_2_WO_6_ crystal lattice parameters were estimated and values are a = 5.456 Å, b = 16.445 Å, and c = 5.444 Å. Bi_2_WO_6_ exhibits several diffraction peaks at 2θ = 28.06°, 32.61°, 46.90°, 53.22°, 58.02^o^, and 75.81°, respectively, which belong to the planes (131), (200), (202), (133), (262) and (400). The high crystallinity of the sample is also shown by the powerful diffraction peaks. Since no other impurity peaks were observed in pure Bi_2_WO_6_ nanostructure. The Bi_2_WO_6_ crystal grain was calculated by using the Scherrer equation which is mainly between (5–15) nm on the (131), (200), and (202) diffraction peaks as shown in Table 1. In addition, the high intensity of the (131) and (200) peaks in Fig. 1 were related to the increase in the energy of the superficies of Bi_2_WO_6_, which enhances the quality of the crystallinity by reducing the defects in the Bi_2_WO_6_^40^. In the state of Bi_2_WO_6_/MWCNTs nanocomposite, Fig. 1 (blue line), represented the characteristic peaks of the Bi_2_WO_6_/MWCNTs nanocomposite at 2θ = 28.30°, 32.91°, 47.2°, 55.89°, 58.6° and 78.53° related to the (131), (200), (202), (133), and (262) planes of the orthorhombic structure of Bi_2_WO_6_, suggestion that the phase of Bi_2_WO_6_ in Bi_2_WO_6_/MWCNTs nanocomposite has no significant change related to the addition of MWCNTs. However, the addition of low content of MWCNTs (less than 1% wt) may be responsible for the absence of MWCNTs peaks in the synthesized nanocomposite and their relatively lower crystallinity than pure Bi_2_WO_6_^41^. Moreover, the value of the average crystallite size increases due to decreasing the β (the half-peak width becomes narrower) as listed in Table 1. The small amount of MWCNTs inclusion in the nanocomposite was said to have prevented MWCNTs from having an impact on the characteristic diffraction^42^. Additionally, it was discovered that the influence of additional MWCNTs caused the peak intensity to gradually decrease in comparison to the intensity in the pure Bi_2_WO_6_.

**Table 1:** Crystalline size of Bi_2_WO_6_ and Bi_2_WO_6_ /MWCNTs nanocomposite by using hydrothermal method.

Materials	θ (°)	Hkl	FWHM (°)	Grain size (nm)
Pure Bi_2_WO_6_	28.06°	131	1.634	5.01
Before added MWCNTs	32.61°	200	1.337	6.19
	46.90°	201	1.391	6.22
Bi_2_WO_6_/MWCNTs nanocomposite	28.30°	131	1.446	5.66
	32.91°	200	1.020	8.12
	47.20°	201	1.183	7.32

**Figure 2:**
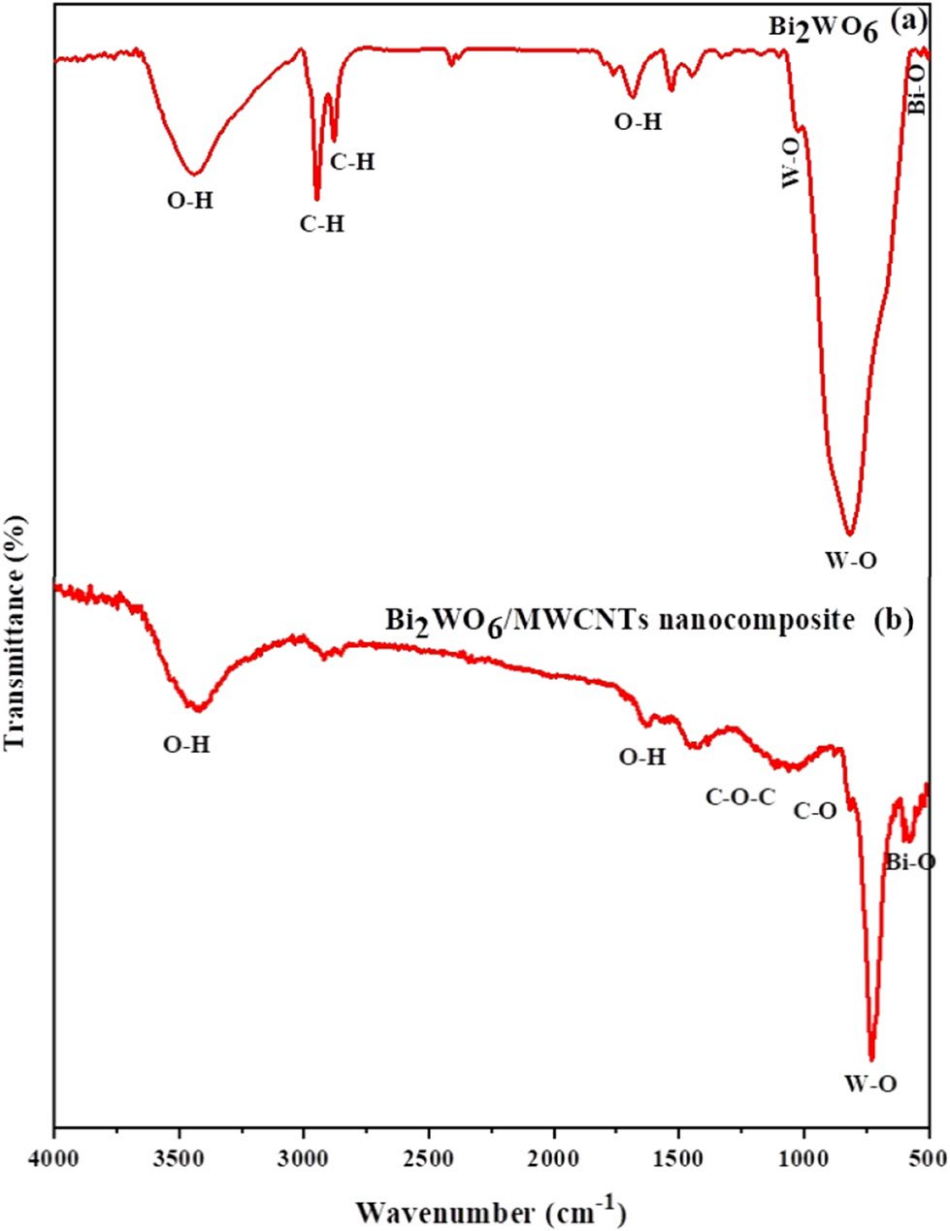
FTIR spectra of (**a**) pure Bi_2_WO_6_ and (**b**) Bi_2_WO_6_ /MWCNTs nanocomposite by hydrothermal method.

The FTIR spectra of pure Bi_2_WO_6_ in the 500–4000 cm^−1^ wavenumber region are shown in Fig. 2a,b at room temperature. The strong bands between 500 and 1300 cm^−1^ were associated with the W–O, Bi–O, and W–O–W stretching vibrations, as illustrated in Fig. 2a^43^. In particular, the W–O bending vibrations mode is corresponding to peak 731 cm^−1^. The Bi–O stretching and deformation vibrations at peak 436 cm^−1^^44^, and the W–O–W stretching at 845 cm^−1^^45,46^, which match earlier papers^47–49^. The stretching vibrations of the -OH groups of adsorbed the H_2_O on the surface of the Bi_2_WO_6_ sample were responsible for the broad absorption band at 3455 cm^−1^. The O–H bond's bending vibration in the sample that was adsorbing water molecules was indicated by the vibration peak at 1624 cm^−1^^50^. The peaks at 2921 cm^−1^ and 2854 cm^−1^ were related to added surfactant-like methyl and methylene groups of CTAB which are consistent with previous papers^51,52^. Furthermore, Fig. 2b displayed the FTIR analysis of the Bi_2_WO_6_/MWCNTs nanocomposite, which exhibited a high and broad peak at 3460 cm^−1^ and a comparatively weak peak at 1622 cm^−1^, respectively. These peaks are ascribed to the O–H stretching vibrations of the water molecules that have been adsorbed on the Bi_2_WO_6_ and MWCNTs^53,54^. After the addition of MWCNTs, the peak at 1622 cm^−1^ becomes weaker, showing that partial surface O–H groups are ingested by the MWCNTs decoration^15^. The bond lengthening or weakening that causes the O–H peak at 3460 cm^−1^ to move to higher wave numbers is caused by an increase in the force constant. This phenomenon happens when the bond length becomes shorter. The modification in the surrounding atom's electronegativity may be the cause of the changing bond length^25^. The main absorption peaks of Bi_2_WO_6_ were observed at (400–800) cm^−1^ which was assigned to the symmetric and asymmetric vibration of W–O at 725 cm^−1^ and the stretching vibration of Bi–O at 584 cm^−1^, respectively^47^. Additionally, the peak of MWCNTs at 1224 cm^−1^ and 1052 cm^−1^ correspond to the C–O–C stretching vibration and the C–O stretching vibration, respectively^55–57^. As indicated in Table 2, the composition of Bi_2_WO_6_ and the presence of a low quantity of MWCNTs were obvious.

**Table 2:** Observed FTIR modes of pure Bi_2_WO_6_ and Bi_2_WO_6_/MWCNTs nanocomposite.

Observed modes (cm^−1^)		
Bi_2_WO_6_	Bi_2_WO_6_/MWCNTs	Attribution
731	725	WO_6_ symmetric stretching
845	–	W–O–W stretching
436	584	Bi–O bending bonds
3455	3460	O–H stretching vibrations
1624	1622	O–H bending vibration
–	1224	C–O–C stretching vibration
–	1052	C–O stretching vibration

**Figure 3:**
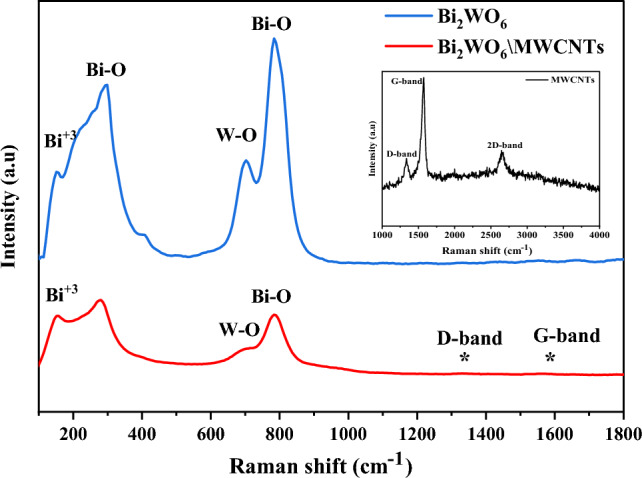
Raman spectra of pure Bi_2_WO_6_ and Bi_2_WO_6_/MWCNTs nanostructures and inset Raman spectra of F-MWCNTs.

The Raman spectra of Bi_2_WO_6_ and Bi_2_WO_6_/MWCNTs nanocomposite are shown in Fig. 3, respectively. The Raman peak positions of pure Bi_2_WO_6_ in the wavenumber range of 200–1000 cm^−1^ correspond to the orthogonal Bi_2_WO_6_ as shown in Fig. 3 (blue line)^58^. These bands can be related to the structure of W–O and Bi–O stretches being visible in the range of (700–800) cm^−1^. Besides, the peak at 703 cm^−1^ exhibits a splatted pattern due to the distortion in W–O interaction owing to size reduction, and the peak at 787 cm^−1^ was related to Bi–O stretches^59^. The band at 295 cm^−1^ could be assigned to translational modes involving simultaneous motions of Bi^+3^ and WO_4_ as a consequence of the addition of CTAB^60^. Similarly, vibrations in the region between (100–450) cm^−1^ for Bi_2_WO_6_ are mainly attributed to rocking or bending modes of the WO_6_ octahedron and BiO_6_ polyhedron. The Bi–O peak at 297 cm^−1^ was split into more prominent bands as a consequence addition of CTAB. Furthermore, it is observed that CTAB prompts a reduction in the thickness and disordered Bi_2_WO_6_ morphological nanostructure. Therefore, it was suggested that the addition of CTAB is responsible for structural alternation that led to forming WO_6_ octahedron^49,61^. Moreover, the Raman spectra obtained agree with the XRD results^62,63^. Figure 3 (red line) displays Raman spectra of Bi_2_WO_6_/MWCNTs nanocomposite. The peak at 703 cm^−1^ was related to W–O interaction and the peak at 787 cm^−1^ belonged to Bi–O stretches^64^. Similarly, to pure Bi_2_WO_6_, the bands relating to Bi rocking and transverse Bi^3+^ motions appeared in the range of (200–400) cm^−1^^65^. When the amount of MWCNTs atoms were embedded into Bi_2_WO_6_, the intensity of the peak at 783 cm^−1^ was reduced compared to pure Bi_2_WO_6_. It might be attributed that some MWCNTs atoms were embedded in the O-W–O lattice and replaced some oxygen atoms that were originally bonded with W. In addition, the two weak peaks around 1595 cm^−1^ and 1346 cm^−1^ for Bi_2_WO_6_/MWCNTs nanocomposite are related to the D band and G band, respectively and the calculated D/G intensity ratio is about 1.1. The D band and G band of F-MWCNTs were about 1332 cm^−1^ and 1571 cm^−1^, respectively as shown in Table 3. The D/G intensity ratio of Bi_2_WO_6_/MWCNTs increased compared with the intensity ratio of MWCNTs (D/G = 0.84). This shifting in intensity ratio towards high value due to the chemical oxidation and increasing D-band intensity was attributed to sp^3^ bonds in F-MWCNTs after the hydrothermal method. Besides, the Raman spectral results reveal that MWCNTs are successfully introduced into the Bi_2_WO_6_ lattice, particularly for ordered and disordered crystal structures of multi-walled carbon nanotubes^58,65^.

**Table 3:** Position and assignation of Raman bands for Bi_2_WO_6_ and Bi_2_WO_6_/MWCNTs nanocomposite synthesized by hydrothermal method.

Material	Raman bands (cm^−1^)	Assignation
Bi_2_WO_6_ before adding MWCNTs	787	Stretching of Bi–O bonds
	703	Asymmetric stretching of WO_6_
	295	Bending of Bi–O bonds
Bi_2_WO_6_/MWCNTs nanocomposite	783	Stretching of Bi–O bonds
	703	Asymmetric stretching of WO_6_
	787	Bending of Bi–O bonds
	1346	*D-band of MWCNTs
	1595	*G-band of MWCNTs
	1332	D-band of MWCNTs
	1571	G-band of MWCNTs

**Figure 4:**
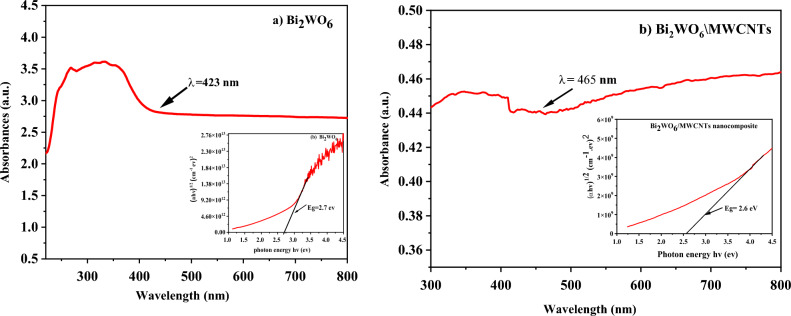
Optical absorbance spectra of (**a**) Bi_2_WO_6_ and (**b**) Bi_2_WO_6_/MWCNTs and inset figures plot of variation of (αhυ)^1/2^ vs. photon energy for Bi_2_WO_6_, and Bi_2_WO_6_/MWCNTs.

In Fig. 4a,b, the UV–Vis absorption spectra of pure Bi_2_WO_6_ and Bi_2_WO_6_/MWCNTs nanocomposite synthesized by hydrothermal method are displayed, respectively. The UV–Vis absorption spectrum of pure Bi_2_WO_6_ was studied in range (300–800 nm). Figure 4a showed a strong and broad absorption band in the range from the UV region 250 nm to the visible region 450 nm with a strong red shift edge at 423 nm. It can be seen that the Bi_2_WO_6_ behaves as a crisp photo-absorption edge in the visible region, showing that the absorption appropriate to the band gap is caused by the intrinsic transition of the nanostructure rather than the transition from impurity levels to give the absorption edge energy which corresponds to E_g_ = 2.7 eV in the set image. Furthermore, the shrinking band gap of pure Bi_2_WO_6_ is always a result of the wavelength's redshift. In the state of Bi_2_WO_6_/MWCNTs nanocomposite as shown in Fig. 4b, It is found that the band gap of E_g_ = 2.6 eV corresponds to a light absorption edge of around 465 nm for Bi_2_WO_6_/MWCNTs nanocomposite. Figure 4b absorption spectra make it clear that the Bi_2_WO_6_/MWCNTs nanocomposite is shifted to a longer wavelength (i.e. redshift) due to the high visible light absorption that occurs after the addition of a small amount of MWCNTs, which exhibit a clear redshift. Table 4 summarizes the observed band gaps for pure Bi_2_WO_6_ and the Bi_2_WO_6_/MWCNTs nanocomposite.

**Table 4:** The Optical band gaps for Bi_2_WO_6_ and Bi_2_WO_6_/MWCNTs nanocomposite.

Materials	Wavelength (nm)	The energy band gap (eV)
Pure Bi_2_WO_6_ before adding MWCNTs	448	2.7
Bi_2_WO_4_/MWCNTs nanocomposite	465	2.6

Furthermore, after the inclusion of MWCNTs, the energy gap lowers dramatically. This is because of the quantum effect which results in the band gap shrinking and shifts in the absorbance from the valance band to the conduction band towards a long wavelength^64^. Also, at the bottom of the conduction band, new localized levels are formed, and these levels are ready to receive electrons and generate tails in the optical energy gap, and these tails act to reduce the energy gap^66^. Since, a small amount of MWCNTs can alter the light absorption of nanocomposite, so confirming sample purity is always necessary when observing optical changes.

These results also agree with XRD patterns analysis related to the creation of oxygen vacancies and low intensity of nanocomposite during hydrothermal method with the addition of a small number of MWCNTs that can contribute to changes in optical properties of reduction band gap and reduced PL of nanocomposite as reveal in next paragraph.

**Figure 5:**
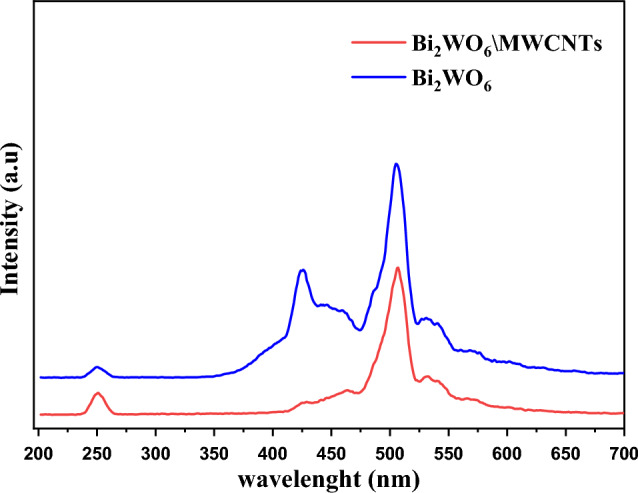
PL spectrum of pure Bi_2_WO_6_ (blue line) and Bi_2_WO_6_/MWCNTs nanocomposites (red line) by hydrothermal method.

The photoluminescence (PL) emission spectra resulted in Bi_2_WO_6_ and Bi_2_WO_6_/MWCNTs nanocomposite samples were used to investigate the charge carrier transfer efficiency and recombination rate of the photogenerated electron–hole (e–h) pairs as well as measurement was carried out to investigate the effect of MWCNTs on the photocatalytic process of semiconductor materials^67^. In Fig. 5, the PL spectrum of each sample had three relatively obvious peaks under the excitation wavelength of 250 nm. As shown in Fig. 5, high emission PL intensity means the rapid charge recombination rate of pure Bi_2_WO_6_ semiconductor photocatalyst and is higher than that of charge carriers of Bi_2_WO_6_/MWCNTs nanocomposite^49,68,69^. Bi_2_WO_6_/MWCNTs reveals low PL intensity refers to a low rate recombination of electron–hole pairs as shown in Fig. 5. These results improved the separation rates of generated (e–h) pairs of nanocomposite and suggest that the addition of MWCNTs can effectively inhibit the recombination rates of electrons and holes. Besides, the results from UV–visible and PL analysis reveal the spectral changes in optical properties result from interfacial electronic interactions, modification of energy bands, and creation of interface transition phase between Bi_2_WO_6_ and MWCNTs providing pathways for charge transfer and separation. The nanocomposite architecture enables synergistic coupling to enhance visible light.

**Figure 6:**
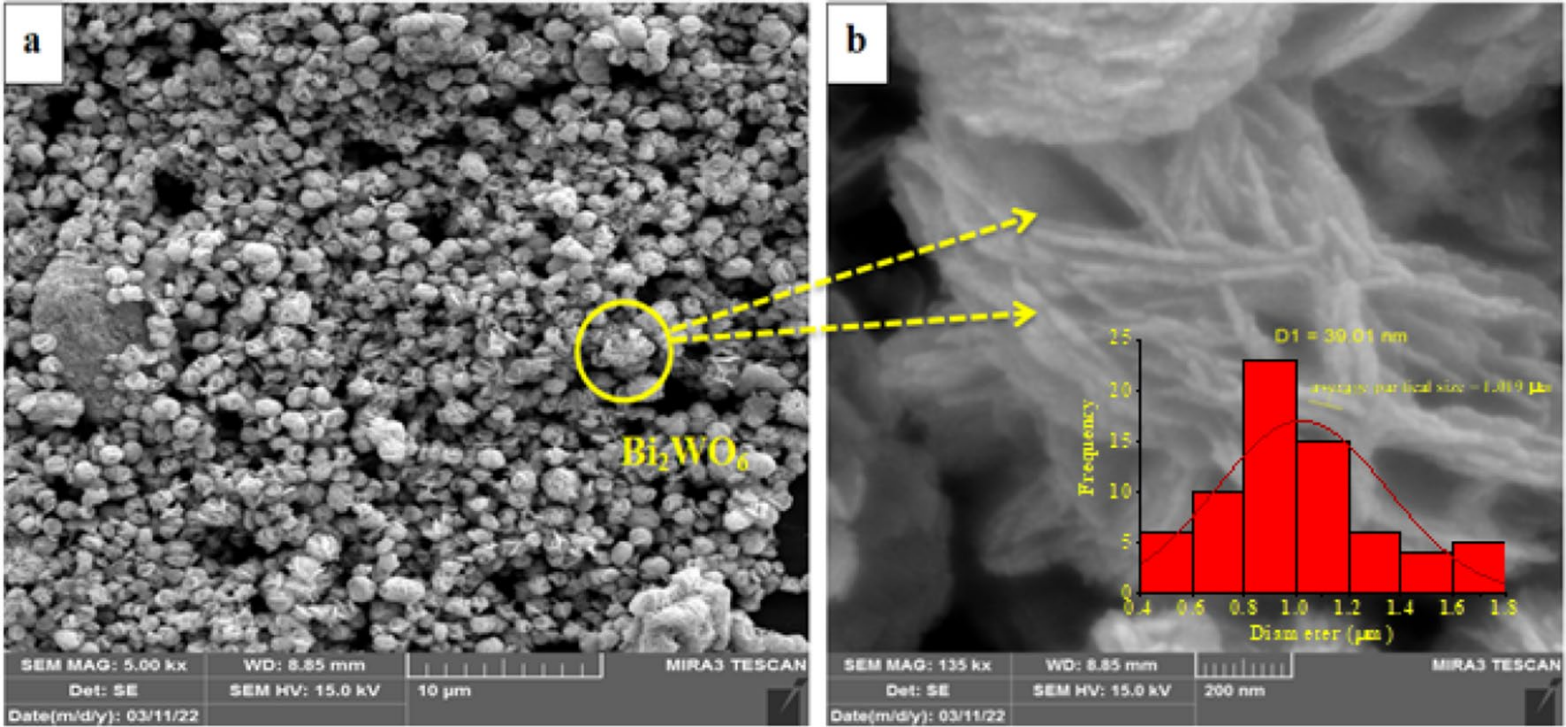
FESEM images of the (**a**,**b**) pure Bi_2_WO_6_ nanostructures and inset image of average particle size.

As demonstrated in Fig. 6a,b, the morphology of synthesized pure Bi_2_WO_6_ and Bi_2_WO_6_/MWCNTs nanocomposite were examined using FESEM analysis, respectively. An expanded view in FESEM revealed that each particle has a flower-like structure made up of a collection of 2D flakes that radiate in all directions as shown in Fig. 6a^70^. Moreover, the high-magnified FESEM image in Fig. 6b, shows that pure Bi_2_WO_6_ was made up of flower-shaped particles with a diameter of 1–2 μm. Moreover, the magnified FESEM in Fig. 6b reveals the crystallization of orthorhombic pure Bi_2_WO_6_. The microstructure of the crystalline nanoparticles then developed into a flower-like shape^71^. Larger particles increased at the expense of the lesser ones, by the Gibbs–Thomson law^72^. Besides, the average particle size distribution of pure Bi_2_WO_6_ nanostructure by using image J was approximately about 1.09 μm as shown in the inset image^73–76^.

Furthermore, the particle size determined by FESEM images exceeds 10 times the particle size predicted by XRD. The layered structure of the Bi_2_WO_6_ nanoparticles would be primarily responsible for the extreme variation^77^. Furthermore, under hydrothermal conditions to yield Bi_2_WO_6_ nanoflakes structures, nucleation processes driven by minimization of the total energy of the system lead to relatively high surface areas with larger sizes^61,77^.

**Figure 7:**
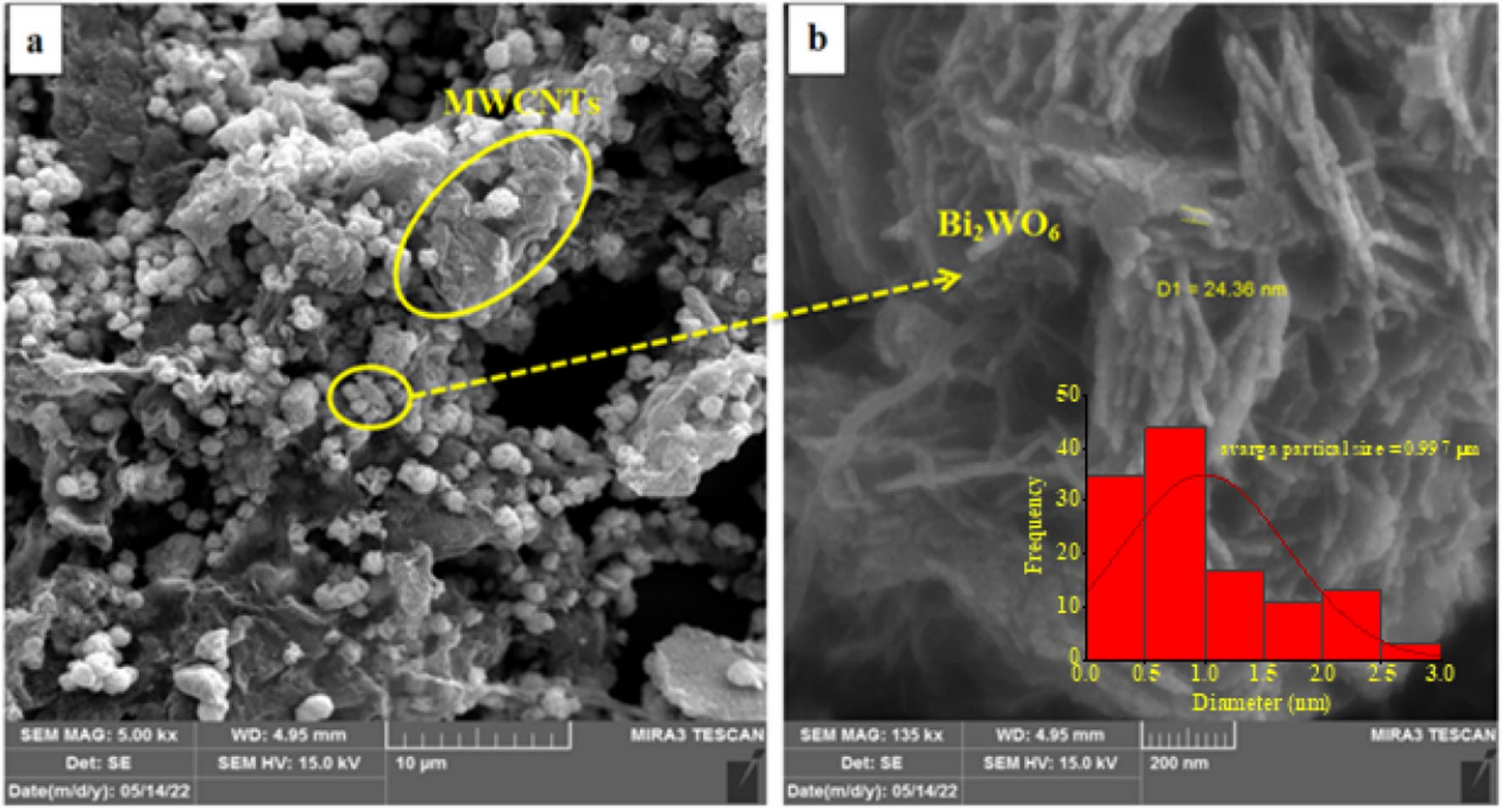
FESEM images of Bi_2_WO_6_/MWCNTs nanocomposite with different magnifications (**a**) at scale bar 10 μm, (**b**) scale bar 200 nm, and inset image of average particle size.

Additionally, the FESEM characterization of Bi_2_WO_6_/MWCNTs nanocomposite reveals a structure similar to the pure flower-like microstructures Bi_2_WO_6_ as shown in Fig. 7a,b. The results showed that the inclusion of a small amount of MWCNTs has a straightforward impact on the morphology of composite made of Bi_2_WO_6_ nanoflakes that have clumped together in Fig. 7b. This finding shows that nanocomposite fabrication is a useful strategy for preventing electron–hole pair recombination^48^. It is revealed that there are nanotubes are stuck to the surface of the micro flowers, and some of them embedded inside the Bi_2_WO_6_ nanoflakes without affecting the structure of Bi_2_WO_6_ nanoflakes and firmly suggesting the existence of MWCNTs in the Bi_2_WO_6_/MWCNTs nanocomposite formed. Moreover, when MWCNTs were added to the pure flower Bi_2_WO_6_, the morphology of the nanocomposite slightly changed which showed that the Bi_2_WO_6_/MWCNTs nanocomposite is made up of nanosheets as shown in Fig. 7b. The FESEM of Bi_2_WO_6_/MWCNTs nanocomposite was exhibited with particle sizes of about 0.997 μm as shown in the inset image of the size distribution histogram. The results reveal that the average particle size of nanocomposite was decreased compared with pure Bi_2_WO_6_ due to F-MWCNTs exhibiting a considerable number of defects and an improved interaction with Bi_2_WO_6_. Additionally, the Bi_2_WO_6_ acts as work pinning centers hindering the MWCNTs grain growth during hydrothermal reaction which leads to the decrease of average particle size.

**Figure 8:**
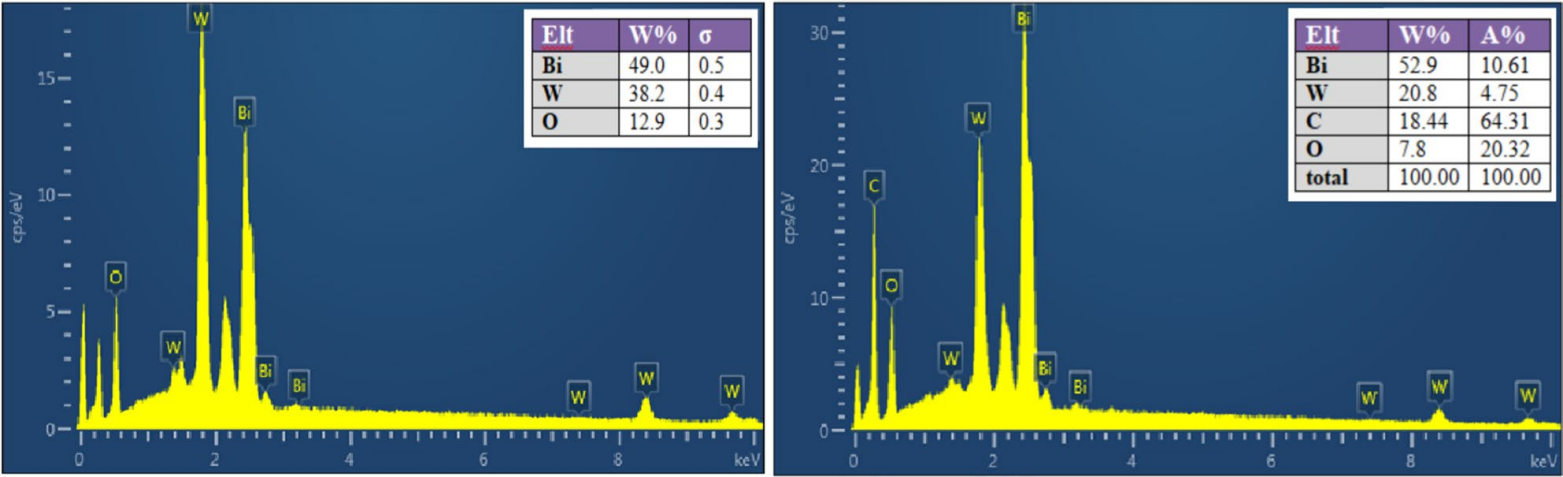
EDS spectrum of the (**a**) pure Bi_2_WO_6_ nanostructures and Bi_2_WO_6_ /MWCNTs nanocomposite (**b**) inset is the table showing the percentage of each component in Bi_2_WO_6_ and Bi_2_WO_6_ /MWCNTs nanocomposite.

The EDS technique was employed to estimate the chemical composition of the resulting flower-like structure of Bi_2_WO_6_ as displayed in Fig. 8a. The results reveal that the product made up of only the Bi, W, and O elements signals occurred in the flower-like structure of Bi_2_WO_6_. To know the chemical composition of Bi_2_WO_6_/MWCNTs nanocomposite, EDS analysis was performed. The results from the EDS spectrum display that the product is collected of C, O, Bi, and W elements corresponding to Bi_2_WO_6_, and the presence of C element due to introducing MWCNTs, Fig. 8b. The element of low content of carbon C in the sample was related to the suitable quantity and moderately low atomic weight of MWCNTs in the nanocomposites with atomic molar ratio Bi:W: C = 2:1:1.

**Figure 9:**
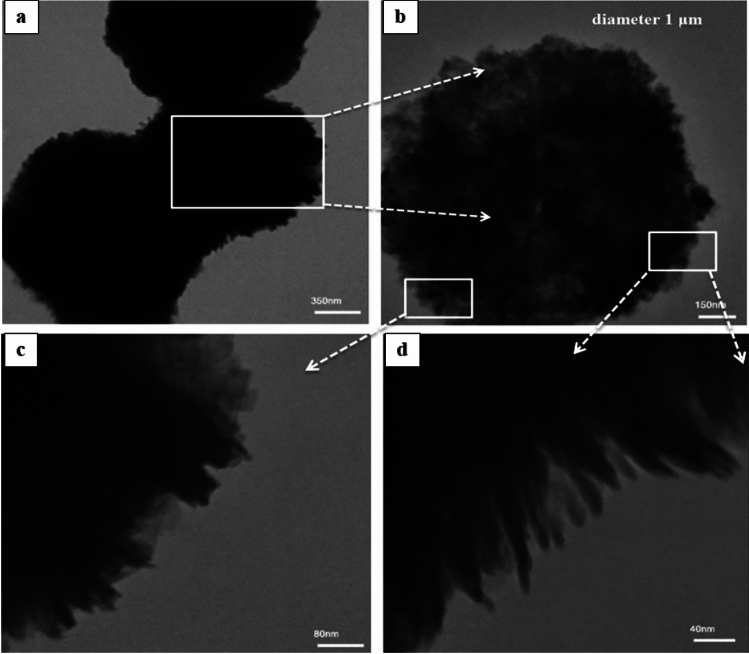
TEM images of the flower-like structure Bi_2_WO_6_ nanostructures, with the different magnifications (**a**) 350 nm, (**b**) 150 nm, (**c**) 80 nm, and (**d**) 40 nm.

**Figure 10:**
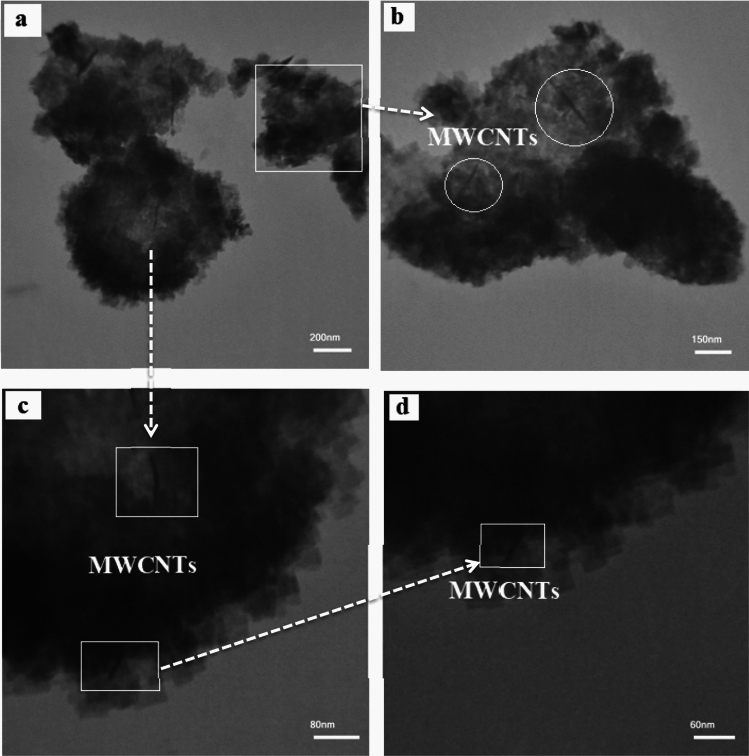
TEM images of the Bi_2_WO_6_/MWCNTs nanocomposite, with the magnifications (**a**) 200 nm, (**b**) 150 nm, (**c**) 80 nm, and (**d**) 60 nm.

More detailed information on the morphology and microstructure of pure Bi_2_WO_6_ nanostructure and Bi_2_WO_6_/MWCNTs nanocomposite was obtained by using TEM at different magnifications in Figs. 9a–d and 10a–d. The TEM images display the whole and individual microspheres of flower-like structure Bi_2_WO_6_ with an average diameter of approximately 1–2 μm respectively, as shown in Fig. 9a,b. As seen in Fig. 9c,d, the magnified image showing the edge indicates that the microsphere was constructed from nanosheets that ranged in size from 100 to 200 nm. Additionally, the nanoplates have a thickness of 20–30 nm^62–65^. The results obtained demonstrate the effective manufacture of pure Bi_2_WO_6_, which is consistent with the XRD analysis.

In the state of synthesized Bi_2_WO_6_/MWCNTs, as shown in Fig. 10a–d, it can be seen that the nanocomposite is composed of typical curly and flimsy nanoplates. Besides, a small amount of nanotubes is embedded inside the Bi_2_WO_6_ nanoflake without affecting the structure. The majority of the area occupying the outer surface is the Bi_2_WO_6_ layer, which becomes irregular after crystallization and occupies most of the space, the results are consistent with the FESEM analysis, which indicates that a low amount of MWCNTs have a small obvious influence on the morphology of Bi_2_WO_6_ composites. It can be observed from Fig. 10c,d at high magnification, that the MWCNTs have been embedded inside the surface of Bi_2_WO_6_ successfully. The surface area of the Bi_2_WO_6_/MWCNTs nanocomposite has increased comparatively due to the relatively small sizes of the nanostructure, which can improve the anticipated electrochemical performance. As the first essential step in the success of any functionalization procedure for the carbon nanotube surface is the treatment (oxidation) of the MWCNTs. Nevertheless, the increase in grain size may be attributed to the greater mobility of incoming reactants and surface diffusion which results in larger crystallites^66^. The above results demonstrate that the Bi_2_WO_6_/MWCNTs nanocomposites are successfully formed indicating that Bi_2_WO_6_ is essentially interacting with MWCNTs nanoparticles^67,68^.

### Antibacterial activity of Pure Bi_2_WO_6_ and Bi_2_WO_6_/MWCNTs nanocomposite

**Figure 11:**
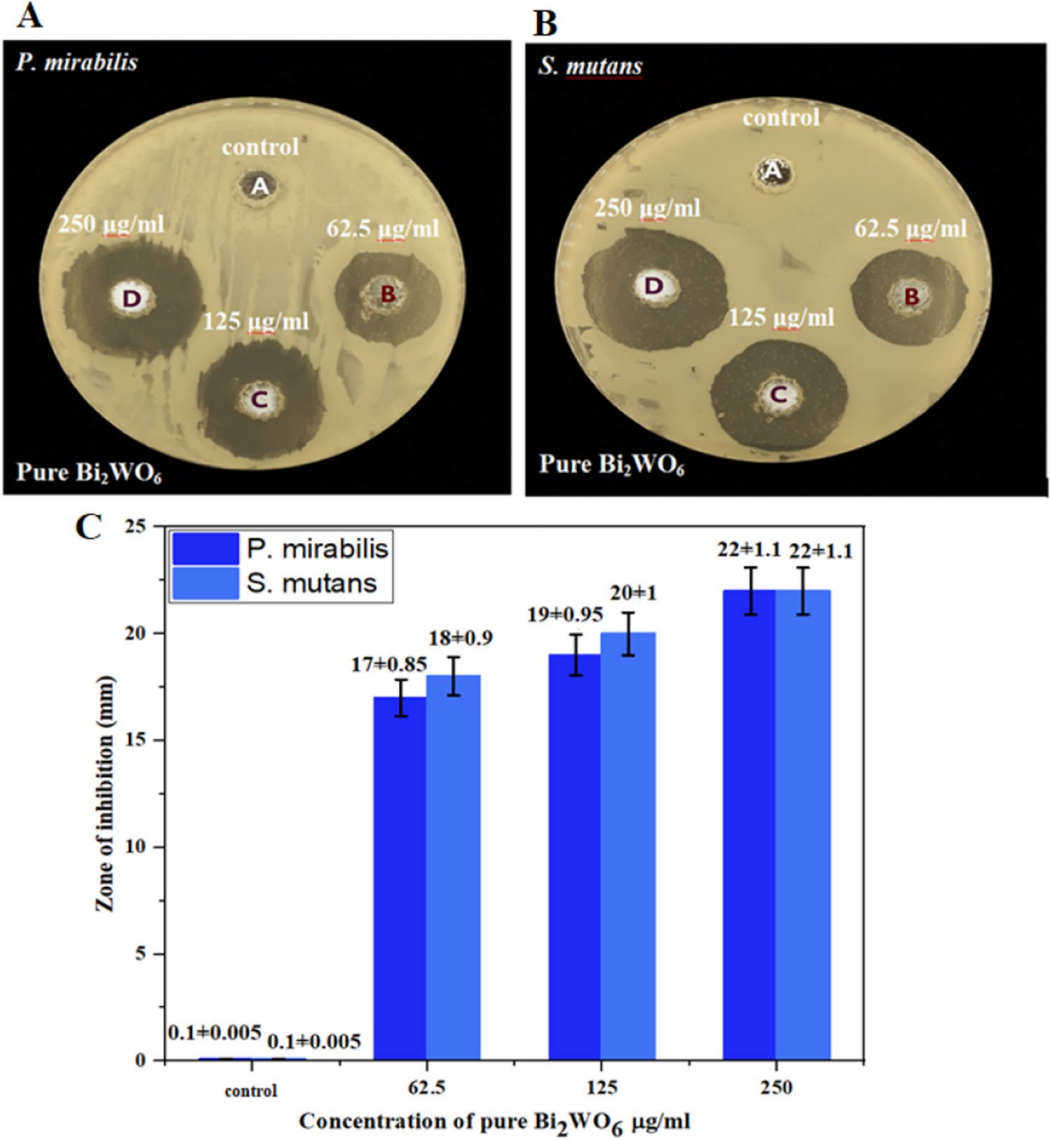
Antibacterial activity of pure Bi_2_WO_6_ nanostructure under visible against (**A**)* P. mirabilis* and (**B**) *S. mutans*. (**C**) Data are represented as mean ± SD of three independent experiments.

**Figure 12:**
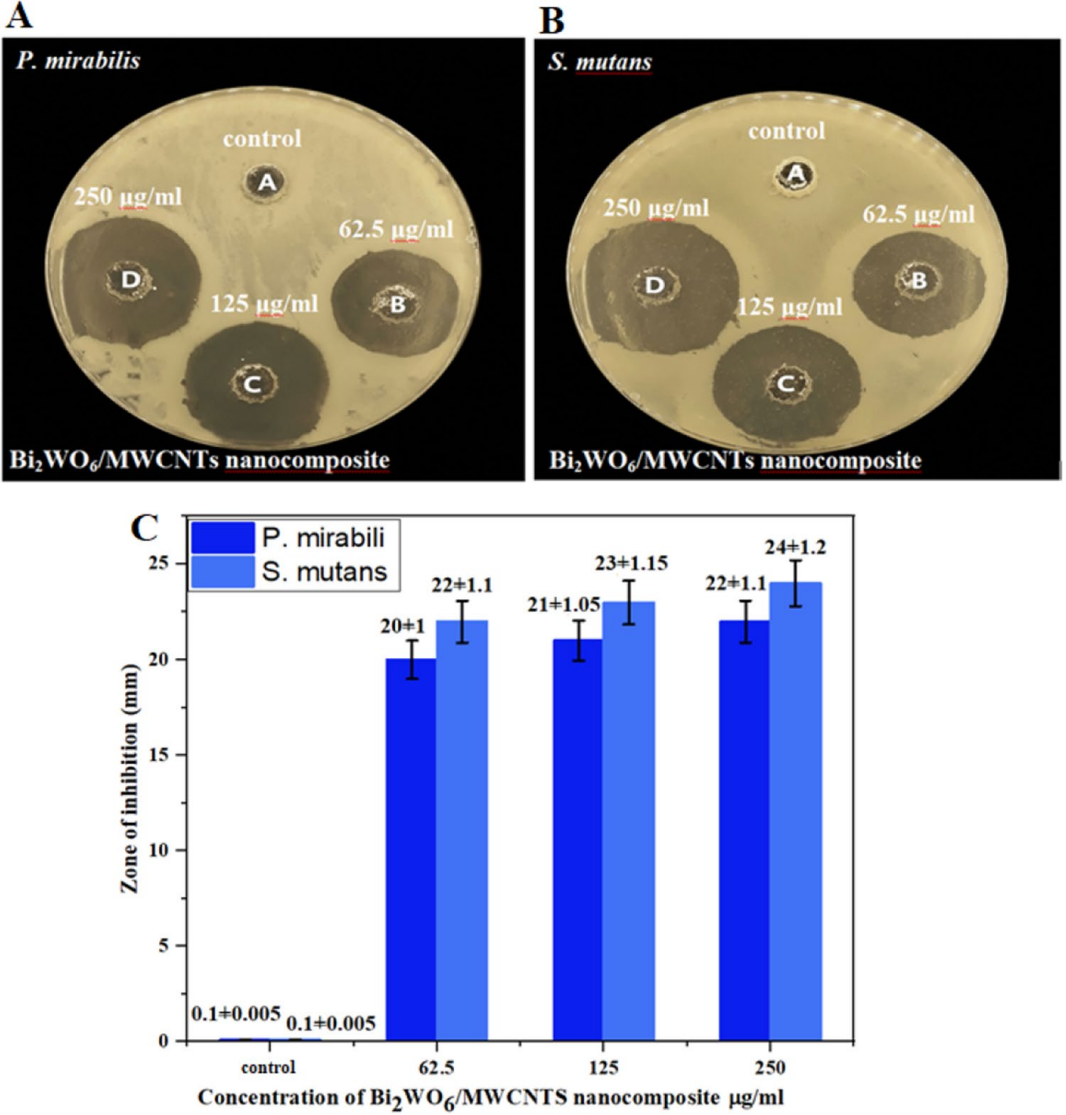
Antibacterial activity of Bi_2_WO_6_/MWCNTs nanocomposite against (**A**) *P. mirabilis* and (**B**) *S. mutans*. (**C**) Data are represented as mean ± SD of three independent experiments.

Agar well diffusion assay is used as indicated in Fig. 11a,b to show the antibacterial impact of pure Bi_2_WO_6_ nanostructure against *P. mirabilis*, a Gram-negative bacterial strain, and *S. mutans*, a Gram-positive bacterial strain. The agar well diffusion assay was performed at various test concentrations (26.5, 125, 250) μg/mL and the bacterial strains were cultured at 37 °C overnight. The inhibition zones against *P. mirabilis* were (17 ± 0.85, 19 ± 0.95, and 22 ± 1.1) mm, and against *S. mutans*, were (18 ± 0.9, 20 ± 1, 22 ± 1.1) mm, as demonstrated in Fig. 11 the inhibition zone is concentration-dependent manner. Since the antibacterial activity of pure Bi_2_WO_6_ was stronger against both bacterial stains. Furthermore, Fig. 11c demonstrates that gram-positive bacteria have a greater inhibitory region than gram-negative bacteria. The antibacterial effect of Bi_2_WO_6_/MWCNTs nanocomposite was investigated using an agar well diffusion experiment at varied concentrations of (26.5, 125, 250) μg/mL. Figure 12a,b and the bar diagram of the antibacterial action Fig. 12c reveal that the inhibition zones increased as the concentrations were raised around (20 ± 1 to 22 ± 1.1 mm) against *P. mirabilis* and (22 ± 1.1 to 24 ± 1.2 mm) against *S. mutans*, respectively. Because their cell walls are more complex, gram-negative bacteria seem to be more resistant than gram-positive bacteria. The cell walls of *S. mutans* bacteria are distinct from those of *P. mirabilis* bacteria. There is only the peptidoglycan (PG) layer on the surface of *S. mutans* bacteria. On the other hand, *P. mirabilis* bacteria have cell walls with outside polysaccharide barriers that protect all polysaccharide structures from degradation^48^ and whose outer surfaces are often resistant to hydrophobic compounds like NPs^69^. Additionally, one of the most crucial elements supporting the antibacterial effect of manufactured Bi_2_WO_6_ nanostructure is their size, which increases their surface area by decreasing their size and enhances their contact with pathogens^70^. The crystallite structure of Bi_2_WO_6_ layered can generate e–h pairs. As mentioned above, these species interact with H_2_O to produce functional groups like OH, H^+^, and O^2^ that can affect the cell surface to break down various parts of the bacterial cell membrane. The loss of function brought on by cell rupture may finally result in cell death in the bacterium^71,72^. Furthermore, the large surface area/volume ratio of the Bi_2_WO_6_ nanostructure promotes interaction between the sample and the bacterial membrane, potentially enhancing adsorption processes^73,78^.

According to the results, pure Bi_2_WO_6_ exhibits lower antibacterial activity against both bacterial strains than nanocomposite materials of Bi_2_WO_6_/MWCNTs. Additionally, the nanocomposite proved to be more effective against *S. mutans* bacteria. This might be because the peptidoglycan layer of *S. mutans* bacteria is thinner than that of *P. mirabilis* bacteria, as illustrated in Fig. 12c, and because there are differences in cell physiology and metabolism^74^. Additionally, it was discovered that nanocomposite can harm bacteria's cell walls structurally and morphologically. Furthermore, the interaction between nanocomposite and bacteria's surface leads to the formation of reactive oxygen species (ROS), which can put the bacteria under oxidative stress and cause the leakage of mitochondrial enzymes and proteins that are essential for cell cycle maintenance. The increased charge transfer through the Bi–O–C bond generated by the functional groups (–OH) and (–COOH) of functionalized F-MWCNTs leading to oxygenation of the membrane as seen in FTIR spectrum analysis was also proposed as a potential mechanism. In general, nanoflake-shaped particles of Bi_2_WO_6_ combined with MWCNTs have smaller average particle sizes (0.997 μm) as compared with pure Bi_2_WO_6_ due to F-MWCNTs exhibiting a considerable number of defects which increases oxygen vacancies and causes the creation of ROS. This causes oxidative stress in the cell by producing (e–h) Paris through a chemical process^75^. The antimicrobial activity of MWCNTs nanocomposite is possibly due to their small size which provides a larger surface area to assist the microbial membrane damage. Further, CNTs induce oxidative stress which plays a further role in antimicrobial mechanisms^79^. Haung et al. examined the mechanical effects that influenced the antimicrobial properties of MWCNTs, such as low wear rates, low friction coefficients, favorable tribological characteristics, and high corrosion resistance^80^. Chen et al. show that the MWCNTs played an important role as “nanodarts” which penetrated bacterial cell walls, reduced membrane potential, caused the release of genetic materials (DNA and RNA), and finally damaged the bacterial cell wall membrane^81^. The stand-out property sp^2^ of carbon-based nanostructures like MWCNTs, reveals amazing electronic structures that result in semiconductivity and metallic of MWCNTs. Besides, the previous work reveals that the metallic property of MWCNTs results in higher antibacterial activity related to the electronic effect as investigated by Vecitis et.al.^82^.

However, several mechanisms could explain how CNTs act as an antibacterial agent such as an increase in ROS generation, attachment of MWCNTs on the microbial cell surface to stimulate the transmembrane electron transfer and induce cell wall and membrane damage, protein dysfunction, and DNA damage when MWCNTs penetrating bacterial cells^83^.

**Figure 13:**
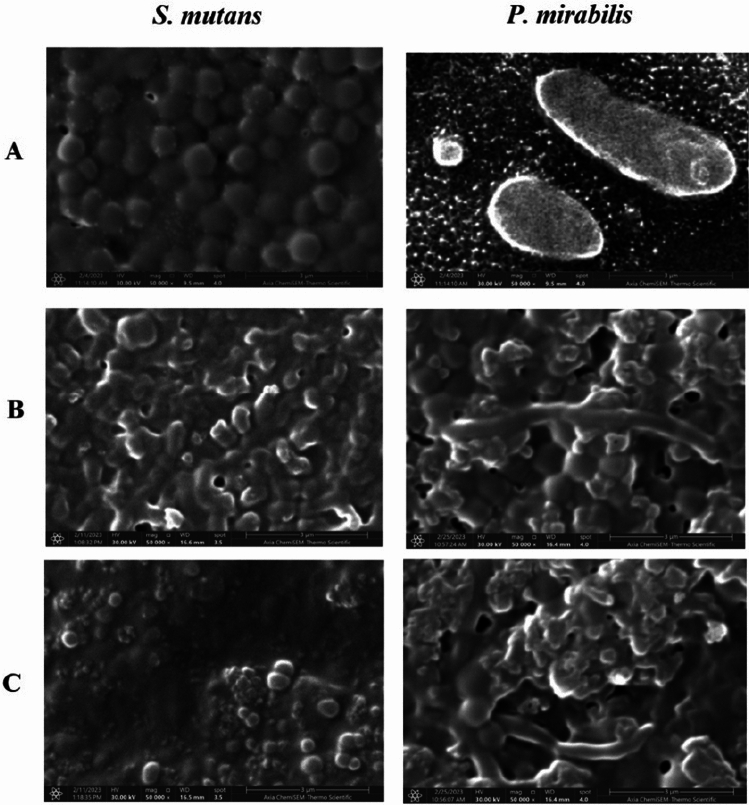
SEM images visualized the morphological effect of pure Bi_2_WO_6_ and Bi_2_WO_6_ /MWCNTs on bacterial strains of *S. mutans* and *P. mirabilis*. The bacterial strains showed changes in the cell membranes like damaged, blabbed, and clumped membranes. Un-treated control bacterial strains (**A**). Bacterial strains treated with pure Bi_2_WO_6_ (**B**). Bacterial strains treated with Bi_2_WO_6_ /MWCNTs (**C**).

The SEM images as shown in Fig. 13A–C, have been acquired to observe the cell deformation upon interaction with pure Bi_2_WO_6_ and Bi_2_WO_6_/MWCNTs, respectively, and the degradation integrity of cells as well as displayed how *S.mutans* and *P. mirabilis* membranes change after being exposed to samples after 24 h of treatment. In Fig. 13A, both untreated (control) cells displayed the typical prokaryotic cell size and intact and independent cell structures. The gram-positive *S. mutans*, with coccus-shaped bacteria, typically appear in clusters but they can also be found in singles or combined. While the gram-negative *P. mirabilis* colonies of rod-shaped with complete cell wells were validated by SEM image as can be seen in Fig. 13A. In the state of cells exposed to pure Bi_2_WO_6_ and Bi_2_WO_6_/MWCNTs, respectively, the SEM images display altered cell morphology that resulted in the flattening and deformation of cell membranes in Fig. 13B,C. Moreover, some of the cells exhibited morphological changes as a result of treatment with pure Bi_2_WO_6_ and Bi_2_WO_6_/MWCNTs, respectively. A few of the treated cells were thinner, slightly smaller in size, and less filled than the untreated cells. Numerous MWCNTs were frequently shown to aggregate with bacterial cells. According to certain investigations, MWCNTs' antibacterial properties depended on having direct contact with cells. These results suggest that after treatment with nanostructures, the cells were cruelly deforming and parts of bacterial covering and intracellular content were missing^19^.

### Bacterial biofilm inhibition

**Figure 14:**
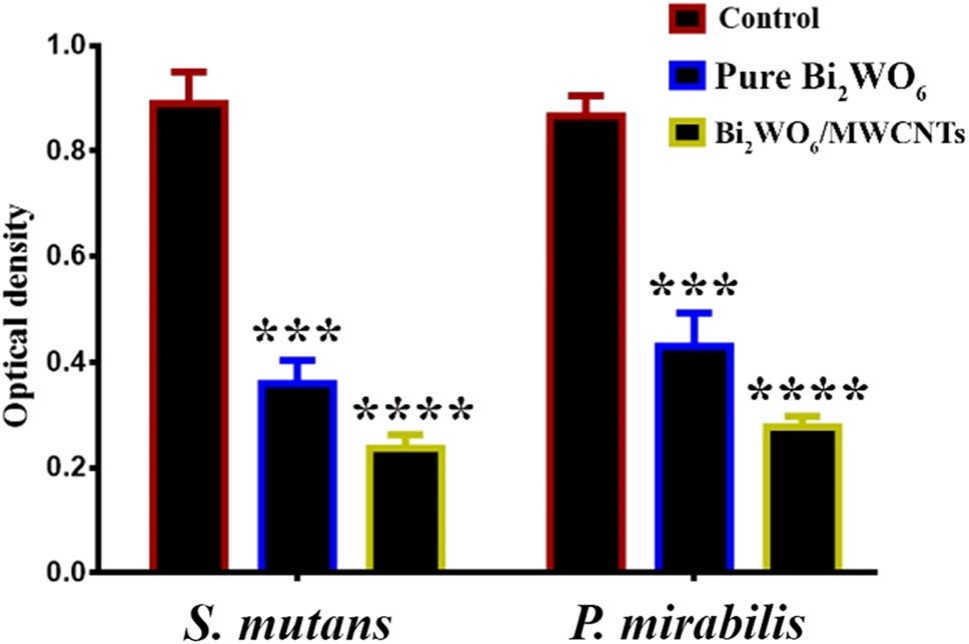
Pure Bi_2_WO_6_ and Bi_2_WO_6_/MWCNTs reduce biofilm formation in bacterial strains. Data are represented as mean ± SD of three independent experiments.

Figure 14 shows how pure Bi_2_WO_6_ and Bi_2_WO_6_/MWCNTs nanocomposites are capable of preventing P. mirabilis and S. mutans from forming biofilms. The creation of biofilms was an essential step in the start of any infection. Bacterial strain adherence to a surface, which occurs through both specific and nonspecific cell-surface interactions, is a necessary first stage in the creation of biofilms. By using a crystal violet stain on the adhering bacterial cells, these biofilms can be identified. Figure 14 illustrates how well pure Bi_2_WO_6_ and Bi_2_WO_6_ MWCNTs performed in this test in terms of biofilm production.

According to the findings, the growth of microbiological strains was considerably inhibited by the pure Bi_2_WO_6_ and Bi_2_WO_6_/MWCNTs nanocomposite. The mechanism behind the production of reactive oxygen species (ROS) may be associated with these decreases in biofilm development. These ROS can cause lipids and proteins to peroxide and oxidize, weakening the Gram + ve cell membrane, changing fluid permeability and ion transport, and inhibiting metabolic processes^68^. Furthermore, the cell walls of Gram-positive and Gram-negative bacteria may be disrupted by physical interactions between the cell and the NPs, whether they are direct or electrostatic.

**Figure 15:**
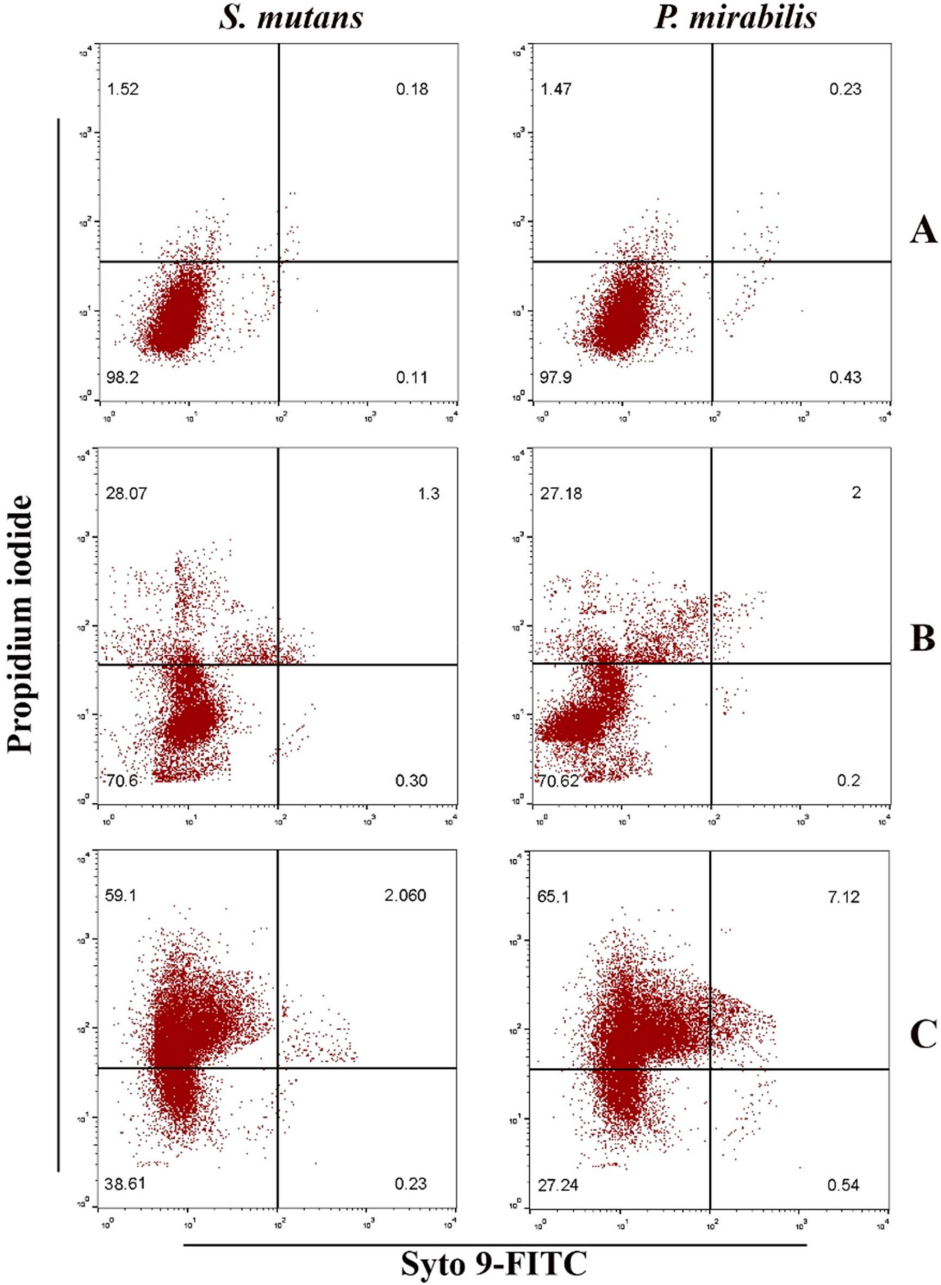
Effect of pure Bi_2_WO_6_ and Bi_2_WO_6_/MWCNTs nanocomposite in metabolic activity in *S. mutans,* and *P. mirabilis* biofilm. (**A**) Control untreated bacterial strain (upper panel) (**B**) Bacterial strains treated with pure Bi_2_WO_6_ (middle panel), and (**C**) Bacterial strains treated with Bi_2_WO_6_/MWCNTs (lower panel).

Dot plots of the *S. mutans* and *P. mirabilis* biofilms assessed by flow cytometry are shown in Fig. 15. Using excitation/emission fluorescence Syto 9 and propidium iodide stains, this assay enabled the differentiation between live and dead cell populations. It was utilized to evaluate metabolic activity in the *S. mutans* and *P. mirabilis* biofilm formed for 48 h. The percentage of live *S. mutans* in the untreated control bacterial strain, as shown in (Fig. 15 upper panel), was 98.2%. similarly, the percentage of live *P. mirabilis* was 97.2%. After treatment with Bi_2_WO_6_ and Bi_2_WO_6_/MWCNTs nanocomposite at a concentration of 125 μg/mL, this percentage decreases to 70.6% and 70.62% when the bacterial strain is treated with pure Bi_2_WO_6_ as in (Fig. 15 middle panel). The percentage of live cells was 38.36% and 27.24% when the bacterial strains were treated with Bi_2_WO_6_/MWCNTs nanocomposite, as shown in (Fig. 15 lower panel). When it came to the reduction of living cells, the Bi_2_WO_6_/MWCNTs nanocomposite performed better than pure Bi_2_WO_6_.

### Anticancer activity of pure Bi_2_WO_6_ and Bi_2_WO_6_/MWCNTs nanocomposite against liver cancer cells

**Figure 16:**
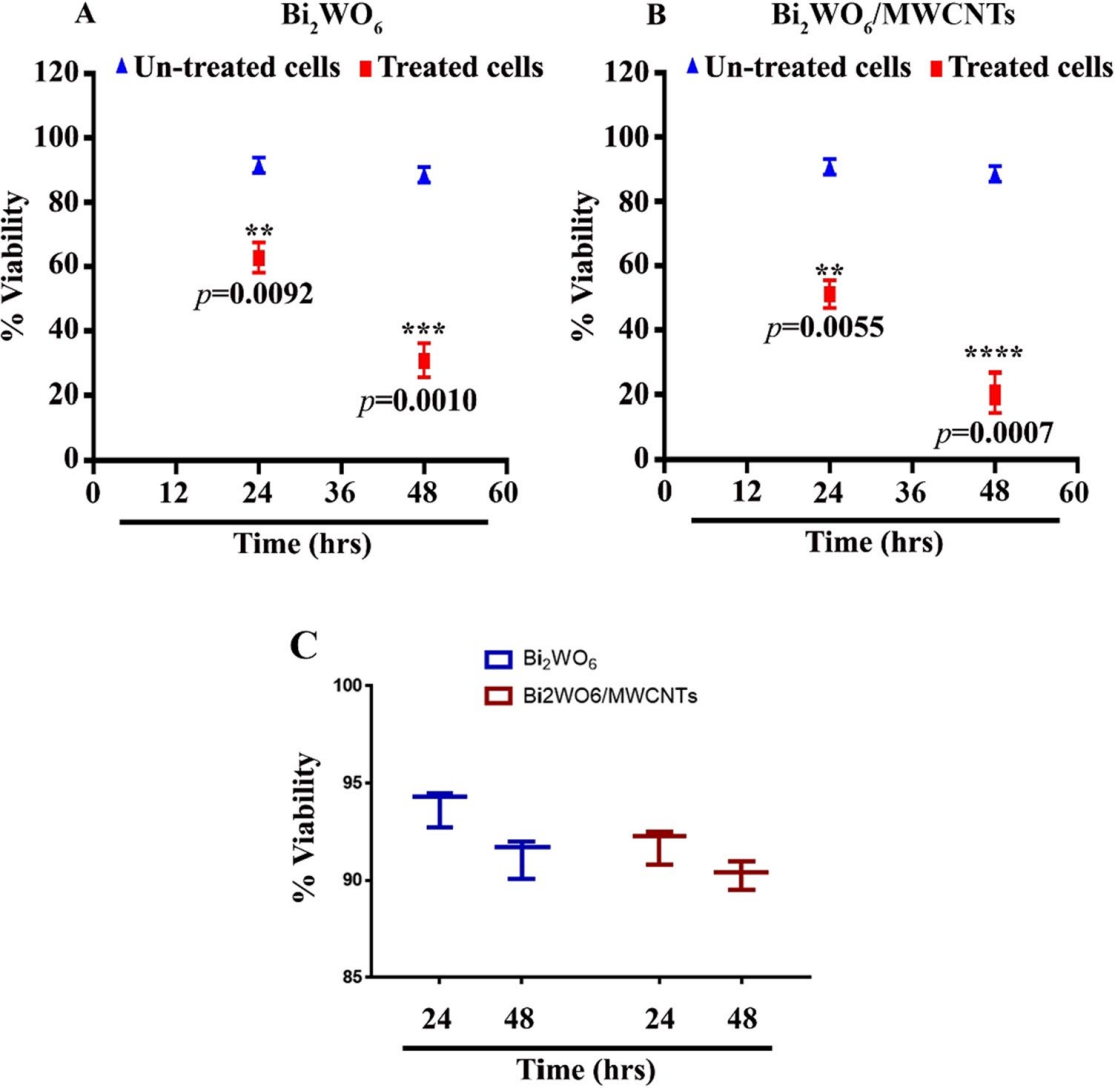
Cytotoxic effect of Bi_2_WO_6_ and Bi_2_WO_6_/MWCNTs NPs in liver cancer cells. (**A**) Bi_2_WO_6_. (**B**) Bi_2_WO_6_/MWCNTs nanocomposite. (**C**) Cytotoxicity of B, Bi_2_WO_6_/MWCNTs nanocomposite in normal cell line (REF) cells.

After 24, and 48 h of treatment with Bi_2_WO_6_ and Bi_2_WO_6_/MWCNTs nanocomposite, liver cancer cells were subjected to an investigation to determine the ability of Bi_2_WO_6_ and Bi_2_WO_6_/MWCNTs nanocomposite to inhibit growth inhibition and proliferation. This was done to investigate the inhibitory impact of Bi_2_WO_6_ and Bi_2_WO_6_/MWCNTs nanocomposite in comparison to the control untreated cells, Bi_2_WO_6_ and Bi_2_WO_6_/MWCNTs nanocomposite suppressed cell viability in a time-dependent manner, as shown in Fig. 16. The cytotoxicity effect of Bi_2_WO_6_/MWCNTs nanocomposite against normal cell line (REF) cells showed a low percent of dead cells as indicated in Fig. 16C. As a result of this study suggested that the pure Bi_2_WO_6_ and the Bi_2_WO_6_/MWCNTs nanocomposite caused cell death. Many studies demonstrated the ability of functionalized CNTs to destroy cancer cells. To treat gastric cancer, Taghavi et al.^84^, created PEGylated SWCNTs that carried DOX and Bcl-xL-specific short hairpin RNA (shRNA). These treatments demonstrated increased gastric cancer cell death due to the synergistic therapeutic action of DOX and shRNA. By using the interaction between CD44 receptors expressed on tumor cells and cholic acid-derivatized HA (CAHA) wrapped around semiconducting single-walled carbon nanotubes (SWCNTs), Bhirde et al. designed a CNT-based DDS by self-assemble method to actively deliver anticancer drug DOX to tumor site^85^. The conjugation of cisplatin and EGF to the surface of oxidized SWCNTs by Bhirde et al. gave the nanosystem the capacity to target targets actively—the link between EGFR overexpression on tumor cells and EGF for increased cisplatin antitumor effectiveness^86^. Though there are still some issues to be resolved, we are optimistic that carbon nanotubes (CNTs) are very promising nanotools with excellent research and significant clinical potential in the treatment of a variety of types of cancer.

### Pure Bi_2_WO_6_ and Bi_2_WO_6_/MWCNTs induce apoptosis in liver cancer cells

**Figure 17:**
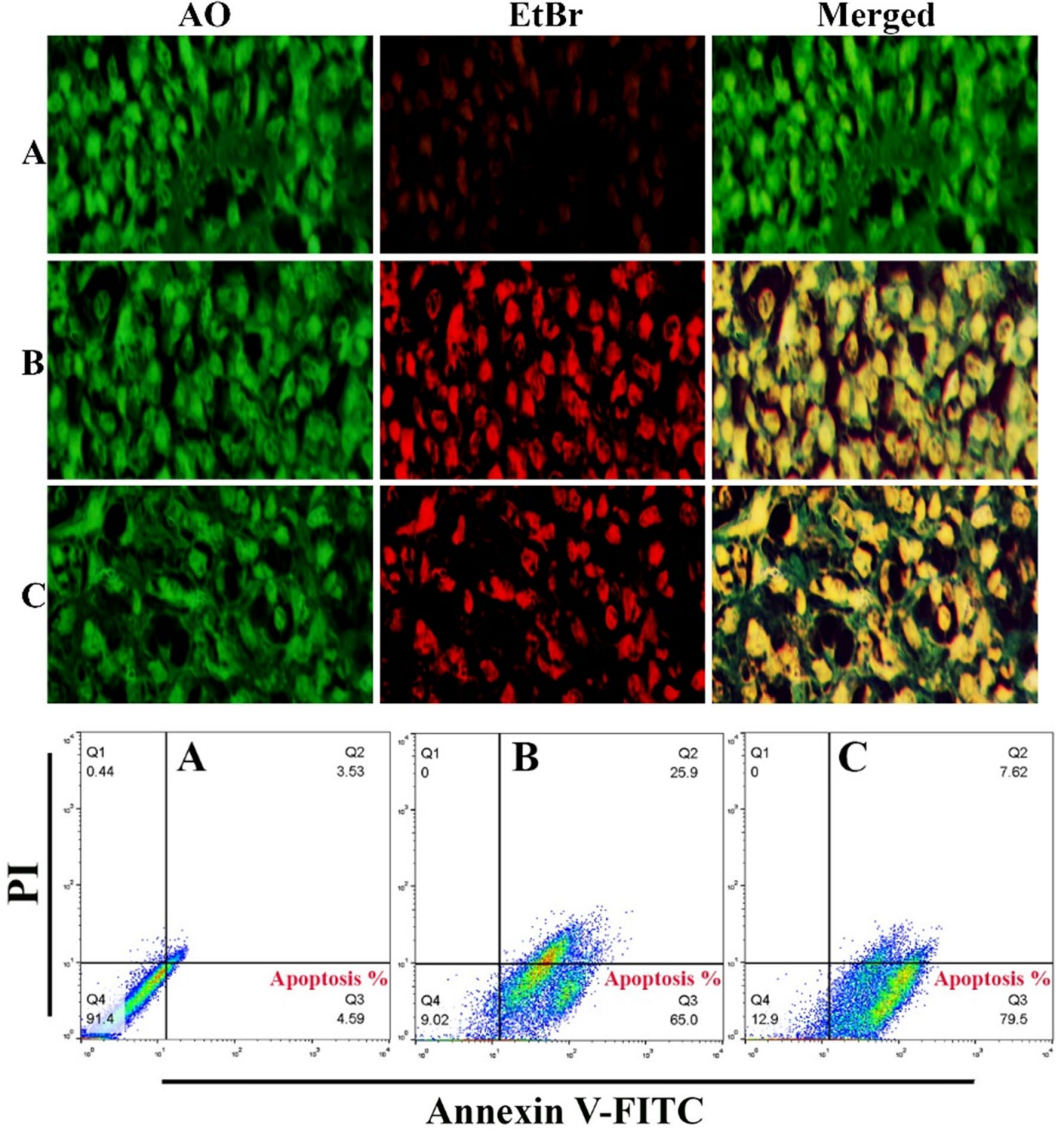
Pure Bi_2_WO_6_ and Bi_2_WO_6_/MWCNTs nanocomposite induces apoptosis in liver cancer cells. (Upper panel) represented AO/EtBr double staining assay. (Lower panel), represented Apoptosis marker (Annexin V) using flow cytometry assay. (**A**) Control untreated cells. (**B**) Cells treated with Bi_2_WO_6_. (**C**) Cells treated with Bi_2_WO_6_/MWCNTs nanocomposite.

The nuclear morphology of the treated cells was examined using a dual staining method consisting of acridine orange and ethidium bromide. DNA damage was used as the criterion for evaluating apoptotic cells. Under the scope of this study, a look was also taken at how effective the Bi_2_WO_6_ and the Bi_2_WO_6_/MWCNTs nanocomposites were. The AO-EB staining was utilized so that the various apoptotic characteristics of the nuclear changes could be investigated. After being stained with AO-EtBr, cells that had not undergone apoptosis were green in color, whereas apoptotic cells had an orange or red color as indicated in Fig. 17 (upper panel). As can be seen in Fig. 16 (upper panel), the cells that were treated with Bi_2_WO_6_ and Bi_2_WO_6_/MWCNTs nanocomposite had many more apoptotic cells than the control cells that had not been treated. Using flow cytometry to label the cancer cells with annexin V-FITC, the proportion of apoptotic cells was identified to confirm the present findings. According to the flow cytometry data, annexin V-FITC was used to identify the cells in quadrant Q3 that were going through apoptosis. Dot plots of Hep-G2 cells treated with pure Bi_2_WO_6_ and Bi_2_WO_6_/MWCNTs nanocomposite for 24 h at an IC_50_ concentration are displayed in Fig. 16 (lower panel). In the control untreated Hep-G2 cells, the majority (93.9%) of cells were viable and non-apoptotic, and in Hep-G2 treated with pure Bi_2_WO_6_ and Bi_2_WO_6_/MWCNTs nanocomposite was a decreased in the viable cells and increased in cells undergoing apoptosis. The percentage of apoptotic cells in the control untreated Hep-G2 was 4.59%. While in pure Bi_2_WO_6_ and Bi_2_WO_6_/MWCNTs nanocomposite-treated Hep-G2 cells, the percentage increased to 65%, and 79.5% respectively. One of these mechanisms is that they act as oxidative stimuli, which in turn promotes inflammation and DNA damage^87^. When cells were treated with pure Bi_2_WO_6_ and Bi_2_WO_6_/MWCNTs nanocomposite, the results of our study showed that the viability of Hep-G2 cells was significantly reduced. Bisht et al. showed evidence that high dosages of ZnO-Fe3O4 magnetic composite nanoparticle induced a cytotoxic impact in human breast cancer cell line (MDA-MB-231) but did not induce this effect in normal mouse fibroblast (NIH 3T3)^88^.

### Pure Bi_2_WO_6_ and Bi_2_WO_6_/MWCNTs induce ROS generation in liver cancer cells

**Figure 18:**
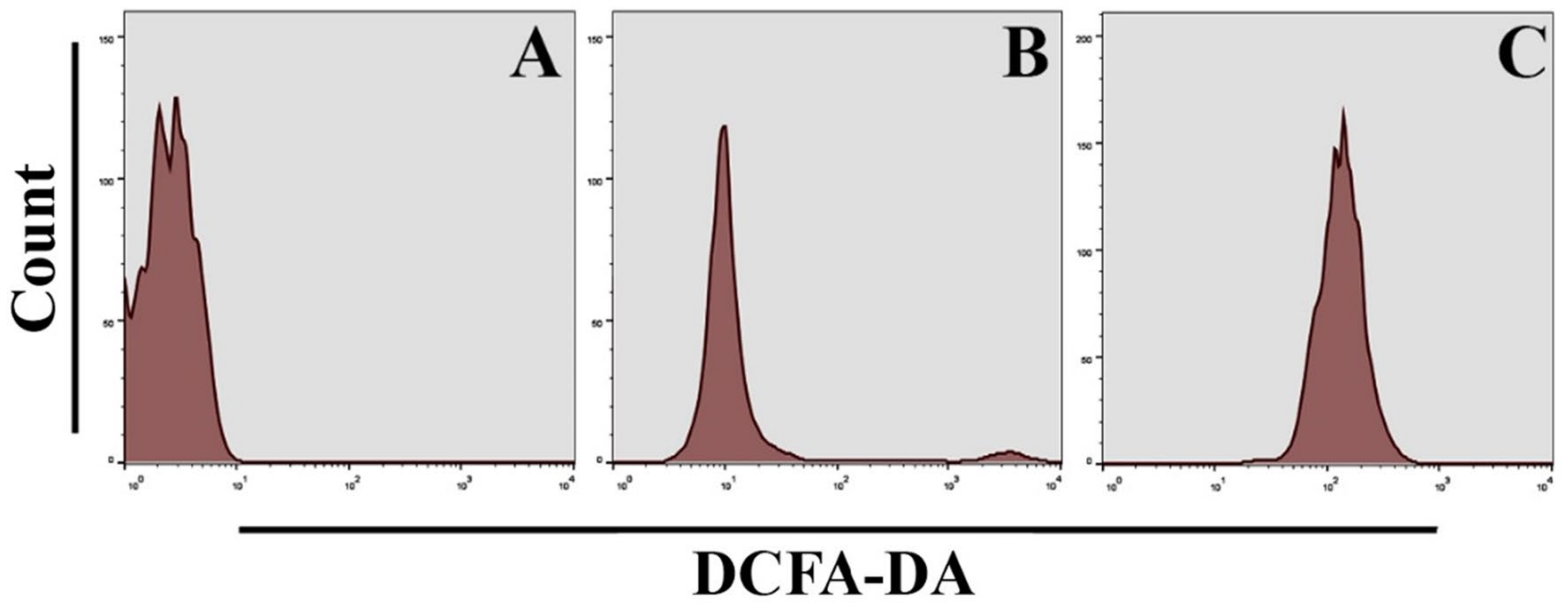
Pure Bi_2_WO_6_ and Bi_2_WO_6_/MWCNTs nanocomposite induce ROS generation in Hep-G2 cancer cells. (**A**) Control untreated cells. (**B**) Bi_2_WO_6_ exposed cells. (**C**) Bi_2_WO_6_/MWCNTs nanocomposite exposed cells.

A considerable increase in the generation of reactive oxygen species was seen in cells treated with Bi_2_WO_6_ and Bi_2_WO_6_/MWCNTs nanocomposite. The fluorescence signal resulting from ROS was found to be higher when compared to the control untreated cells. The buildup of reactive oxygen species (ROS) in liver cancer cell lines after treatment with Bi_2_WO_6_ and Bi_2_WO_6_/MWCNTs nanocomposite was investigated in the current study. An increase of ROS was seen in cells that had been treated with Bi_2_WO_6_ and Bi_2_WO_6_/MWCNTs nanocomposite. ROS levels were measured with a DCFH-DA probe, as demonstrated in Fig. 18. When the liver cancer cells were treated with Bi_2_WO_6_ and Bi_2_WO_6_/MWCNTs nanocomposite, the results revealed that the level of reactive oxygen species (ROS) was enhanced.

### Pure Bi_2_WO_6_ and Bi_2_WO_6_/MWCNTs cause mitochondrial dysfunction in liver cancer cells

**Figure 19:**
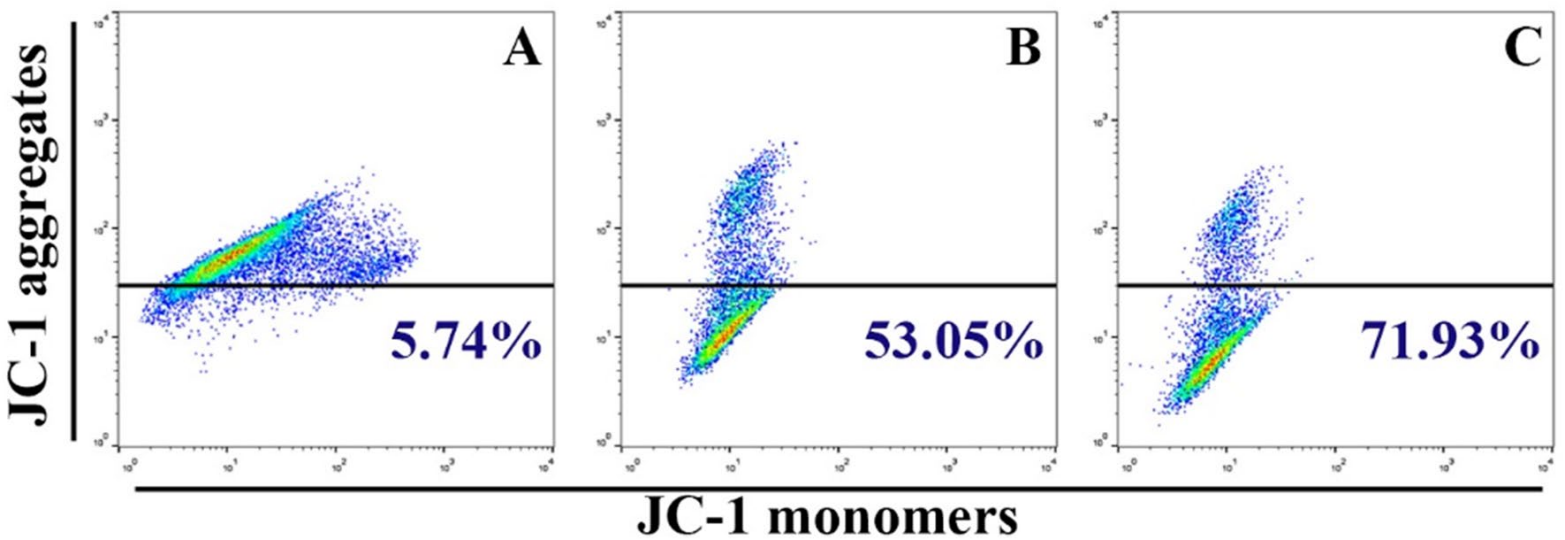
Pure Bi_2_WO_6_ and Bi_2_WO_6_/MWCNTs nanocomposite reduce mitochondrial membrane potential in Hep-G2 cancer cells. (**A**) Control untreated cells, (**B**) Bi_2_WO_6_ exposed cells, and (**C**) Bi_2_WO_6_/MWCNTs exposed cells.

**Figure 20:**
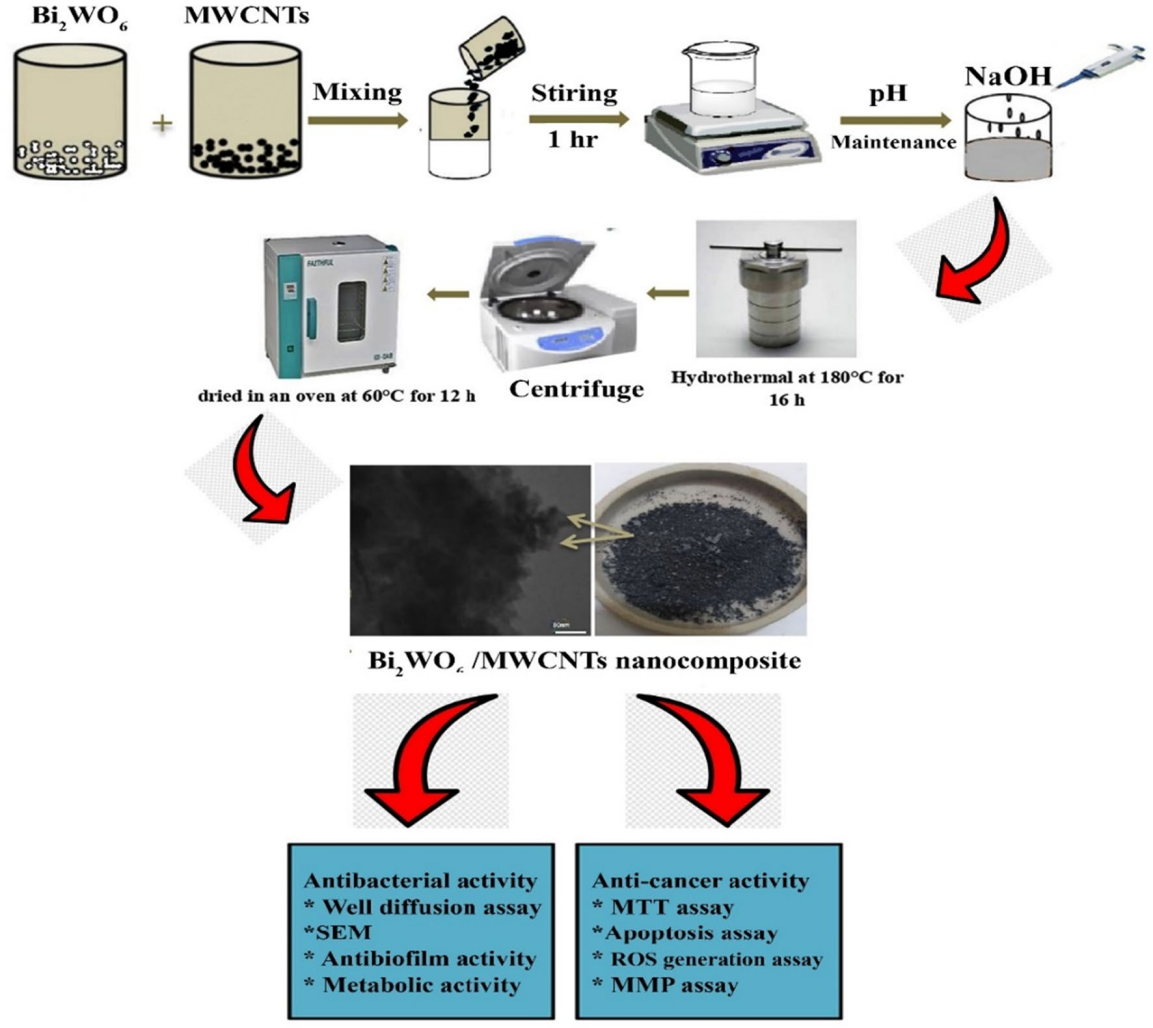
Preparation of Bi2WO6/MWCNTs nanocomposite and evaluation of their antibacterial and anticancer activities.

Hep-G2 cells were stained with JC-1 to determine which mitochondria were healthy and which were damaged. On liver cancer cell lines, the impact of the Bi_2_WO_6_ and Bi_2_WO_6_/MWCNTs nanocomposite is assessed. To determine whether or not mitochondrial damage has occurred, it is known that mitochondrial membrane potential (∆Ψm) is produced by the proton pump of the electron transport chain, which is a component that is required for the production of ATP. For this reason, we additionally assessed MMP using JC-1 staining. As indicated in Fig. 19, the promotion of JC-1 monomers increased noticeably depending on the type of treatment that is given for liver cancer cell lines. According to the findings presented above, the treatment of liver cancer cells Hep-G2 is accompanied by a disruption of the oxidative balance in cancer cells as well as an impairment of protective anti-oxidative molecules. This causes cells to be subjected to excessive oxidative stress and mitochondrial dysfunction which leads to the subsequent release of cytochrome c which causes the activation of caspases-9 and caspase-3 pathways. Preparation of Bi_2_WO_6_/MWCNTs nanocomposite and evaluation of their antibacterial and anticancer activities are presented in Fig. 20.

## Conclusion

In this study, pure Bi_2_WO_6_ and Bi_2_WO_6_/MWCNTs nanocomposite were successfully fabricated using a hydrothermal approach. The characterization methods demonstrated that Bi_2_WO_6_ retained its orthorhombic structure and the introduction of small amounts of MWCNTs had little impact on the morphology, structure, optical, and composition of Bi_2_WO_6_ flower-like structure as demonstrated using FESEM, EDX TEM, XRD, FTIR, and Raman. Besides, this configuration enables the nanocomposite to take advantage of the high adsorption capacity and large specific surface area of MWCNTs, leading to the formation of additional active sites as revealed by FESEM and TEM. The addition small amount of MWCNTs led to significant quantum confinement-induced band gap narrowing of Bi_2_WO_6_ in the nanocomposite. Additionally, the Bi_2_WO_6_/MWCNTs nanocomposite's band gap energy drops to 2.6 eV, which is consistent with the light absorption range shifting to the red region and the associated reduced band gap energy. The Bi_2_WO_6_/MWCNTs nanocomposite's PL spectrum improved the rate at which carriers separated, leading to the formation of more active sites and an increase in adsorption capacity.

Moreover, pure Bi_2_WO_6_ and Bi_2_WO_6_/MWCNTs nanocomposite reveal its antibacterial effects against *P. mirabilis* and *S. mutans* bacteria and demonstrated antibacterial efficacy and biofilm formation, enhancing its properties in comparison to pure Bi_2_WO_6_. SEM analysis and biofilm test further reveal the efficiency of Bi_2_WO_6_/MWCNTs nanocomposite in cell destruction process with cell membrane envelope and spreading to the intracellular as well as demonstrated the dimension dependence of Bi_2_WO_6_/MWCNTs and enhancing its properties compared with pure Bi_2_WO_6_. Generally, the results of the current study showed that the Bi_2_WO_6_ and Bi_2_WO_6_/MWCNTs nanocomposite considerably reduced bacterial biofilm formation. Also, Bi_2_WO_6_/MWCNTs nanocomposite acts as cytotoxic and apoptosis inducer in liver cancer cells via modulates ROS pathway. Furthermore, these results recommend that pure Bi_2_WO_6_ and Bi_2_WO_6_/MWCNTs nanocomposite could be promising materials for developing antimicrobial and cancer agents.

## Data Availability

The data that support the findings of this study are available from the corresponding author upon reasonable request.
